# Explainable machine learning model and gene expression programming for predicting reinforced concrete beams moment capacity exposed to fire

**DOI:** 10.1038/s41598-025-32100-z

**Published:** 2025-12-15

**Authors:** Pouyan Fakharian, Younes Nouri, Arezoo Asaad Samani, Danial Rezazadeh Eidgahee, Mohammad Reza Torabi, Seyed Rohollah Hoseini Vaez

**Affiliations:** 1 Institute of Research and Development, Duy Tan University, Da Nang, Vietnam; 2 School of Engineering & Technology, Duy Tan University, Da Nang, Vietnam; 3 Department of Civil Engineering, Faculty of Engineering, Ferdowsi University of Mashhad, Mashhad, Iran; 4 Department of Civil Engineering, Faculty of Engineering, University of Qom, Qom, Iran; 5 Department of Civil Engineering, Faculty of Engineering, Lorestan University, Khorramabad, Iran

**Keywords:** Moment capacity, Machine learning, Reinforced concrete beam, Gene expression programming, Fire, Engineering, Materials science, Mathematics and computing

## Abstract

In this study, a new formulation for the moment capacity (*M*_r_) of Reinforced Concrete (RC) beams under fire conditions is estimated using Gene Expression Programming (GEP). In addition, the use of Machine Learning (ML) methods such as XGBoost, AdaBoost, and LightGBM is investigated for estimating the *M*_r_ of RC beams in fire. The database for predicting the *M*_r_ of RC beams includes 280 samples. In this paper, the cross-section width *b*_*w*_, cross-section depth *d*, distance from the beam edge to the center of steel reinforcement *d*_*eff*_, area of steel reinforcement *A*_*st*_, time duration of fire *t*, compressive strength of concrete *f*_*c*_, and moment capacity of the beam under fire *M*_*r*_ are considered as the parameters of ML models. Several statistical metrics were employed to assess the performance of the models, including the mean absolute error (*MAE*), mean square error (*MSE*), root mean square error (*RMSE*), coefficient of determination (*R*^2^), and gradients of regression lines (*k* and *k*′). In this study, Shapley Additive exPlanations (SHAP) analysis was used to interpret the predictions of the XGBoost model, which was selected for its high accuracy with the best R^2^ and the lowest error rate. The results indicate that the methods used demonstrate high accuracy in estimating the *M*_r_ of RC beams.

## Introduction

Fire phenomenon strongly affects the performance of structures. Therefore, fire resistance of structures is a necessity for the engineering community. The use of fire standards for structures exposed to fire can reduce the effects of fire on the structure. Thermal properties that are most affected by temperature include specific heat, thermal conductivity, density, coefficient of thermal expansion, and diffusion. However, the thermal properties of density, specific heat, and thermal conductivity are the most affected and should be considered for thermal analysis^1^. The temperature of a steel member exposed to fire increases and its strength and stiffness decrease, leading to deformation and failure of the steel member^2^. The increase in steel temperature depends on the area of steel exposed to fire, the severity of the fire, and the fire protection applied to the steel. Concrete structures behave more favorably than steel structures when exposed to fire. Concrete is non-combustible and has a low thermal conductivity^3,4^. Reinforced concrete is made up of concrete combined with reinforcing elements such as rebar or fibers. Reinforcing elements are used to compensate for the low tensile strength and ductility of concrete and also resist shear. Under fire conditions, increased temperature greatly affects the behavior of members, resulting in significant thermal gradients in the heated areas. The temperature of the compressive zone of concrete is also strongly affected by the cross-sectional dimensions and fire intensity^5^. Many studies have been conducted to investigate the effects of fire on structures^6–9^.

Cao and Nguyen^10^ evaluated the performance of post-fire RC beams under different fire durations and retrofitting designs. They concluded that fire could alter the dominant flexural failure of RC beams to flexural-shear failure. This change in failure mode under fire conditions is also observed in ultra-high-performance concrete (UHPC) beams^11^. Noman and Yaqub^12^ studied the post-fire curing behavior of RC beams exposed to 900 °C for 1 h. Based on their results, the strength and stiffness of RC beams decreased by 40% and 33% under fire conditions and regained almost 90% of their strength and stiffness after post-fire curing.

Hostetter et al.^13^ used a sacrificial layer of additional reinforcement as a beneficial solution for mitigating fire-induced effects before post-fire curing. Banerji and Kodur^14^ studied the effect of fire on UHPC RC beams using numerical methods. Spalling and temperature-dependent material properties for steel and concrete were considered as variables.

Ren et al.^15^ investigated the effects of hybrid fiber-reinforced Reactive Powder Concrete (RPC) beams under fire conditions. They proposed a Finite Element (FE) simulation-based equation for predicting the fire resistance of hybrid fiber-RPC beams to avoid brittle failure in RC beams. Kodur and Banerji^16^ compared the behavior of RC beams with different concrete strengths under fire. Ultra-high-performance concrete (UHPC), high-strength concrete (HSC), and normal-strength concrete (NSC) were considered, and they concluded that UHPC had lower fire resistance and extensive spalling compared to the other concretes. In UHPC, explosive spalling is a common phenomenon, which can be mitigated by using the new two-stage hot air curing method proposed by Qin et al.^17^. Hybrid BFRPs/steel reinforcements for RC beams were evaluated by Hassan et al.^18^ for enhancing shear strength, crack tolerance, and mitigating brittle failures. They showed that this combination had a positive effect on the mechanical behavior of RC beams with hybrid reinforcements.

Liu et al.^19^ investigated the effects of fire duration and spalling failure variables including depth and local area ratio in RC beams under fire scenarios using experimental and numerical analyses. They established a database of 250 samples for parametric study. The application of ML methods has been used in a limited number of studies. Zhao et al.^20^ used the XGBoost model to predict the fire resistance of concrete-filled steel tubular (CFST) columns. Nariman et al.^21^ proposed an optimization method for designing RC beams for strength and stiffness. They used surrogate modeling based on ML methods for response prediction, and their database was developed using the Box-Behnken method. They achieved good agreement between experimental and numerical results for failure and cracking modes.

Khan et al.^22^ used machine learning methods for predicting the response of fiber-reinforced polymer (FRP) strengthened RC beams. Solhmirzaei et al.^23^ used Support Vector Machine Regression (SVMR) and multigene genetic programming (MGGP) to estimate the flexural capacity of UHPC-RC beams. Kumar et al.^24^ used four ML models including XGBoost, Random Forest (RF), K-nearest neighbors (KNN), and Convolutional Neural Network (CNN) for estimating failure properties of HPC and UHPC RC beams. Naser^25^ used surrogate and nine ML models for evaluating the spalling behavior of concrete under fire conditions. Naser and Kodur^26^ also investigated spalling behavior of fire-induced RC columns using explainable ML models. Panev et al.^27^ used the SVM method for preliminary response prediction of insulation in floor systems under fire scenarios.

Ye et al.^28^ developed an FE-based ML algorithm such as Gradient Boosting (GB) and RF for displacement prediction of structures under fire analysis.

Erdem^29^ investigated the fire performance of RC beams with box cross-sections. Using a novel analytical approach, he divided the cross-section into multiple segments to accurately capture non-uniform temperature distributions and material property changes during fire exposure. In this research, the performance of the structure under fire conditions was evaluated using finite difference methods for temperature analysis and segmental force balance principles for moment capacity calculations.

Erdem^30^ calculated the *M*_r_ of RC beams exposed to fire using artificial neural networks (ANN). In this study, 280 samples with eight parameters were analyzed, and the ANN model was developed for estimating the *M*_r_ of beam. The effect of input variables on the *M*_r_ of the beam was also evaluated. Furthermore, Erdem^31^ proposed a new ANN model using 294 data samples and seven parameters. The new ANN model with high prediction accuracy can estimate the *M*_r_ of reinforced concrete slabs exposed to fire.

Ahmadi et al.^32^ estimated shear stress values in steel fiber reinforced concrete (SFRC) beams without stirrups using new design formulas. The study investigated the parameters of conventional reinforced concrete and fiber reinforced concrete using GEP and ANN models. The proposed formulas predicted the shear capacity of SFRC beams.

Fakharian et al.^33^ developed artificial intelligence models to predict the compressive strength of hollow concrete block masonry prisms. They compared ANN, GEP, and hybrid methods for processing grouped data using 102 samples. The input parameters of their model were the prism height-to-width ratio, mortar strength, and concrete block strength. Sensitivity analysis showed concrete block strength to be the most influential parameter in all models. Chen et al.^34^ used ANN, GEP, and MLR models to predict the compressive strength of steel pipes filled with recycled concrete using 103 samples, and the ANN model demonstrated higher accuracy. Kumarawadu et al.^35^ predicted the fire resistance of FRP-RC beams using XGBoost, CatBoost, LGBoost, and GRB models with an accuracy of more than 92%. The most important parameters affecting fire resistance were loading ratio, cross-sectional area of tension bars, insulation depth, concrete cover thickness, and FRP cross-sectional area. Habib et al.^36^ also predicted the failure potential of FRP-RC beams exposed to fire. They analyzed feature importance to identify key parameters affecting structural failure. Wang et al.^7^ presented a genetic-evolutionary approach to evaluate the fire resistance of FRP-RC beams. The LightGBM model predicted the fire resistance time and the deformation at the moment of failure with high accuracy. Ho et al.^37^ evaluated the fire resistance and spalling of reinforced concrete columns using machine learning. The results showed that the AdaBoost model performed best with an accuracy of 87% in predicting spalling and R^2^ = 0.96 in predicting fire resistance. The study by Hao et al.^38^ showed that if the data is well prepared and processed, the choice of algorithm type has less impact on the final result.

This study presents a systematic comparative analysis between powerful and novel boosted tree algorithms (XGBoost, AdaBoost, LightGBM) and a symbolic evolution model (GEP) in the problem domain. Unlike pure black box models, this study provides a practical solution for engineers by extracting a simple and applicable mathematical equation from the GEP model (with an accuracy of R^2^ = 0.97) and interpreting and clarifying its results by applying SHAP analysis to the best model (XGBoost with an accuracy of R^2^ = 0.9906). This research empirically demonstrates how the combination of feature engineering and hyperparameter optimization can achieve accuracy that is on par with or exceeds previous reference models. Since the effect of fire on RC beams has not been studied using GEP, XGBoost, AdaBoost, and LightGBM algorithms, one of the aims of this study is to examine these methods for estimating the moment capacity of RC beams under fire conditions. SHAP analysis was performed on the XGBoost model, which demonstrated high accuracy and low error. The mean|SHAP| and feature importance values were also calculated for the XGBoost model. The database for estimating the *M*_r_ of RC beams includes 280 samples, and the cross-sectional geometry, material properties, and fire duration were selected as evaluation parameters. The Bayesian hyperparameter method was used to optimize the models, and their prediction accuracy was calculated using statistical measures and regression analysis.

## Moment capacity of the RC beam under fire exposure

To calculate the moment capacity (*M*_*r*_) of a reinforced concrete (RC) beam subjected to fire, several key parameters must be evaluated. These include the duration of fire exposure, material strength, temperature rise, and thermal distribution across the beam section. Once these factors are established, the tensile and compressive forces within the beam’s cross-section can be computed. The moment capacity is calculated when force equilibrium is achieved under fire conditions. The ISO834 standard provides the temperature-time curve for fire exposure as^39^:

1 $$T=345{\mkern 1mu} {\log _{10}}(8t+1)+{T_{ambient}}\;(^\circ {\mathrm{C}})$$

where temperature (*T*) is a function of time, *T*_*ambient*_ is the ambient temperature in normal condition, and *t* is the fire duration (in minutes).

### Material strength reduction at elevated temperatures

At elevated temperatures, the tensile strength of reinforcement steel and the compressive strength of concrete significantly decrease. According to Eurocode 2^40^, the reduced tensile strength of steel (*f*_*suT*_) at elevated temperatures can be determined as follows:

2 $$\frac{{{f_{suT}}}}{{{f_{su\:20\:^\circ {\mathrm{C}}}}}}={k_s}\,\,\,\,\,,\,\,\,\,\,\,\,\,if\,\,\,\left\{ {\begin{array}{*{20}{c}} {0 \leqslant T \leqslant 350} \\ {350 \leqslant T \leqslant 700} \\ {700 \leqslant T \leqslant 1200} \\ {1200 \leqslant T} \end{array}} \right.\,\,\,\, \Rightarrow \,\begin{array}{*{20}{c}} {{k_s}=1} \\ {\,\,\,{k_s}=1.899 - 0.00257T} \\ {{k_s}=0.24 - 0.0002T} \\ {{k_s}=0} \end{array}$$

where *f*_*su*20°C_ is the tensile strength at 20 °C and *k*_*s*_ is the reduction factor of tensile strength. Similarly, the compressive strength of concrete (*σ*_*cT*_) at elevated temperatures can be calculated as^40^:

3 $$\frac{{{\sigma _{cT}}}}{{{\sigma _{c\:20\:^\circ {\mathrm{C}}}}}}={k_c}\,\,\,\,,\,\,\,\,\,\,\,\,if\left\{ {\begin{array}{*{20}{c}} {T \leqslant 100\;} \\ {100 \leqslant T \leqslant 400} \\ {400 \leqslant T \leqslant 900} \\ {900 \leqslant T} \end{array}} \right.\,\,\,\,\, \Rightarrow \,\,\,\,\,\,\begin{array}{*{20}{c}} {{k_c}=1} \\ {{k_c}=(1.067 - 0.00067T)} \\ {{k_c}=(1.44 - 0.0016T)} \\ {{k_c}=0} \end{array}$$

where *σ*_*c*20°C_ is the compressive strength of concrete at ambient temperature (20 °C), and *k*_*c*_ is the reduction factor of compressive strength.

### Temperature distribution across the Cross-section of beam

The thermal gradient across the beam’s cross-section is determined by solving the heat conduction equation. The two-dimensional steady-state heat conduction problem, assuming constant thermal conductivity and no heat generation, is governed by Eq. (4)^41^ and can be solved analytically for simple geometries with well-defined boundary conditions. However, for more complex cases, numerical methods such as the finite element, boundary element, or finite difference approaches are typically employed. In this study, the finite difference method is used to numerically solve the steady-state heat conduction equation in Cartesian coordinates, employing a square mesh discretization as described in Eq. (5)^30^:

4 $$\frac{{{\partial ^2}T}}{{\partial {x^2}}}+\frac{{{\partial ^2}T}}{{\partial {y^2}}}=0$$

5 $${T_{left}}+{T_{right}}+{T_{top}}+{T_{bottom}} - 4{T_{node}}=0$$

### Bending moment of RC beam under fire

**Fig. 1:**
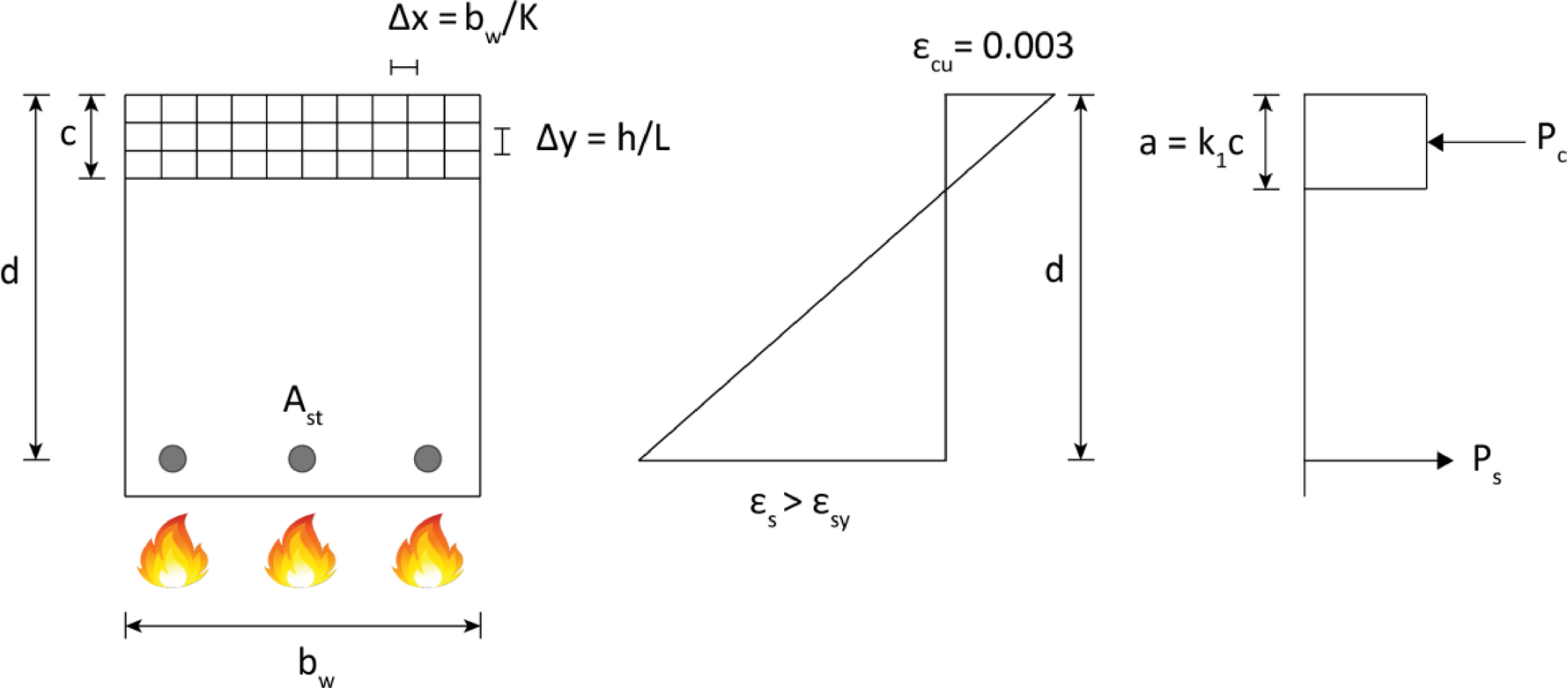
Stress and strain diagrams for concrete beam with rectangular section.

After determining the temperature field, the mechanical properties of each subdivided region (*K×L mesh*) are evaluated to compute the moment capacity of beam. The tensile force of the reinforcement can be calculated as^30^:

6 $${P_s}=\mathop {\mathop \sum \nolimits^{} }\limits_{{i=1}}^{K} \mathop {\mathop \sum \nolimits^{} }\limits_{{j=1}}^{L} {k_{sij}}{f_y}{A_{sij}}$$

where *f*_*y*_ is the yielding strength of steel at 20 °C; *k*_*sij*_ is the reduction factor for each mesh of steel. The total compressive force in the RC beam is obtained by summing the individual force contributions from all elements in the compression zone, which can be calculated using Eq. (7)^30^:

7 $${P_c}=0.85\mathop {\mathop \sum \nolimits^{} }\limits_{{i=1}}^{K} \mathop {\mathop \sum \nolimits^{} }\limits_{{j=1}}^{{\frac{a}{{{\mathrm{\boldsymbol{\Delta}}}y}}}} {k_{{c_{ij}}}}{f_c}{\mathrm{\boldsymbol{\Delta}}}x{\mathrm{\boldsymbol{\Delta}}}y$$

where *f*_*c*_ is the compressive strength of concrete at 20 °C; *k*_*cij*_ is the reduction factor for each mesh of concrete in RC beam; and *a* is the depth of compressive stress block. Finally, the moment capacity of the RC beam can be obtained as^29^:

8 $${M_r}=0.85\mathop {\mathop \sum \nolimits^{} }\limits_{{i=1}}^{K} \mathop {\mathop \sum \nolimits^{} }\limits_{{j=1}}^{{\frac{a}{y}}} {k_{{c_{ij}}}}{f_c}{\mathrm{\boldsymbol{\Delta}}}x{\mathrm{\boldsymbol{\Delta}}}y\left( {d - \frac{{{\mathrm{\boldsymbol{\Delta}}}y}}{2} - j{\mathrm{\boldsymbol{\Delta}}}y} \right)$$

where *d* is the effective depth of beam section. The complete specifications of the RC beam section, as well as stress and strain diagrams, are shown in Fig. 1.

## Database description

**Fig. 2:**
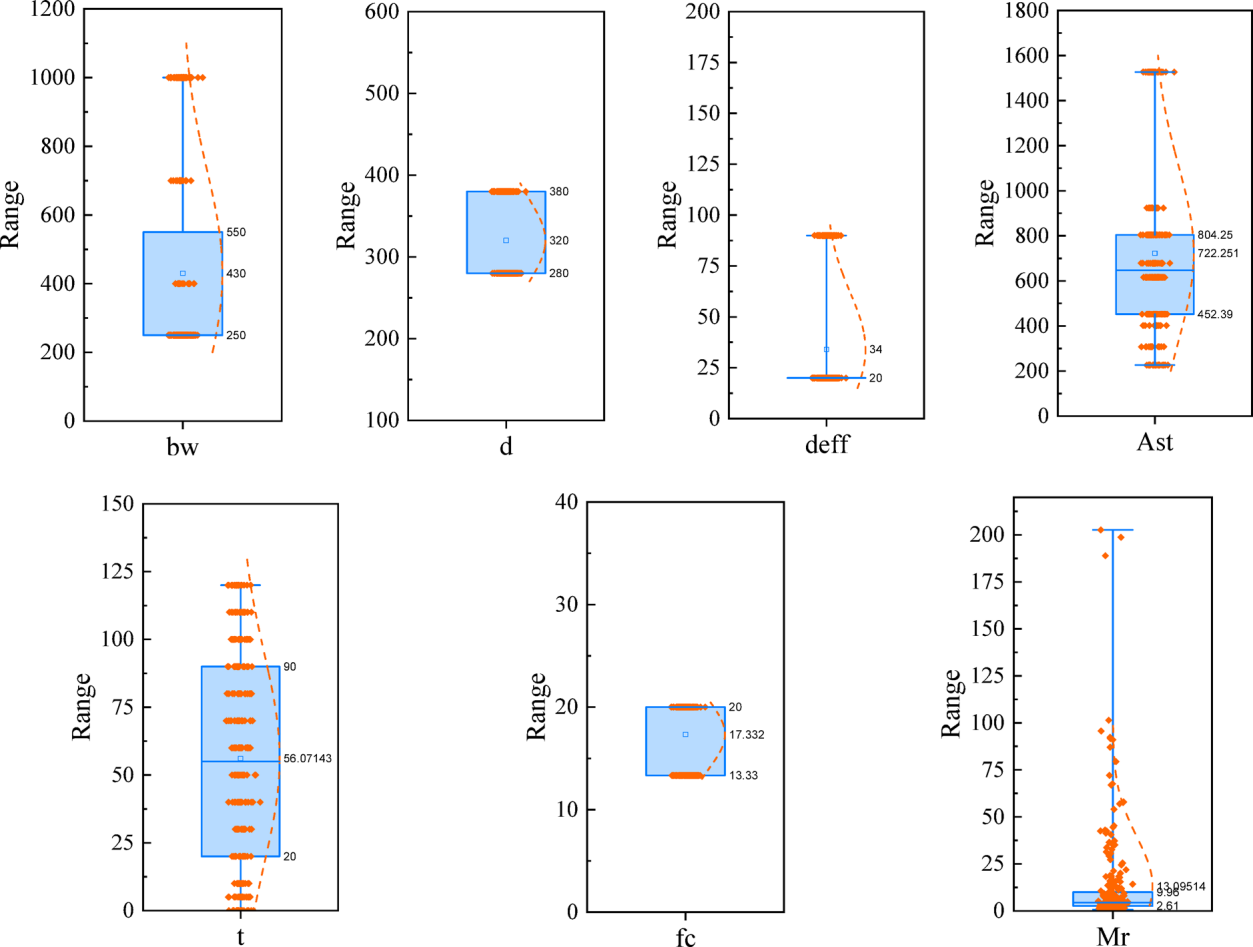
Box plot for inputs and output parameters.

The database for predicting the moment capacity of RC beams under fire conditions includes 280 samples extracted from Erdem^30^. In this paper, the parameters used in the ML models, including the cross-section width$\:{\:b}_{w}$, cross-section depth $\:d$, distance from the beam edge to the center of steel reinforcement $\:{d}_{eff}$, area of steel reinforcement $\:{A}_{st}$, time duration of fire $\:t$, compressive strength of concrete $\:{f}_{c}$, and moment capacity of the beam under fire $\:{M}_{r}$, were investigated. The database was prepared through careful homogenization processes, including ensuring unit consistency across all parameters, identifying and handling potential outliers via statistical analysis, and verifying the physical correctness of the values. Although no artificial data expansion methods were employed, the accuracy and adequacy of the dataset were indirectly confirmed by achieving very high prediction accuracy and consistency with the results of the reference model. The main focus of this study was on careful data preprocessing and the extraction of optimal features based on mechanical principles. In this study, the dataset was divided into 80% for training and 20% for testing. Table 1 presents the specifications of these parameters along with the statistical characteristics for each parameter, including the mean, standard deviation, kurtosis, skewness, minimum, and maximum values. To better determine the range of the variables, box plots of the input and output parameters are shown in Fig. 2. In these figures, the 25th and 75th percentile values, as well as the mean value of each parameter, are indicated. The box plots are drawn based on the range between the maximum and minimum values of the data.

**Table 1:** Statistical properties for inputs and output variables.

Parameter	$\:{b}_{w}$ (mm)	$\:d$ (mm)	$\:{d}_{eff}$ (mm)	$\:{A}_{st}$ (mm^2^)	$\:t$ (min)	$\:{f}_{c}$ (MPa)	$\:{M}_{r}$ (kN.m)
Mean	430.000	320.000	34.000	722.251	56.071	17.332	13.095
Standard Deviation	302.783	49.078	28.050	390.059	38.807	3.273	26.229
Kurtosis	− 0.335	− 1.845	0.276	0.149	− 1.289	− 1.845	26.077
Skewness	1.236	0.410	1.508	0.990	0.101	− 0.410	4.611
Minimum	250.000	280.000	20.000	226.190	0.000	13.330	0.690
Maximum	1000.000	380.000	90.000	1526.810	120.000	20.000	202.630

**Fig. 3:**
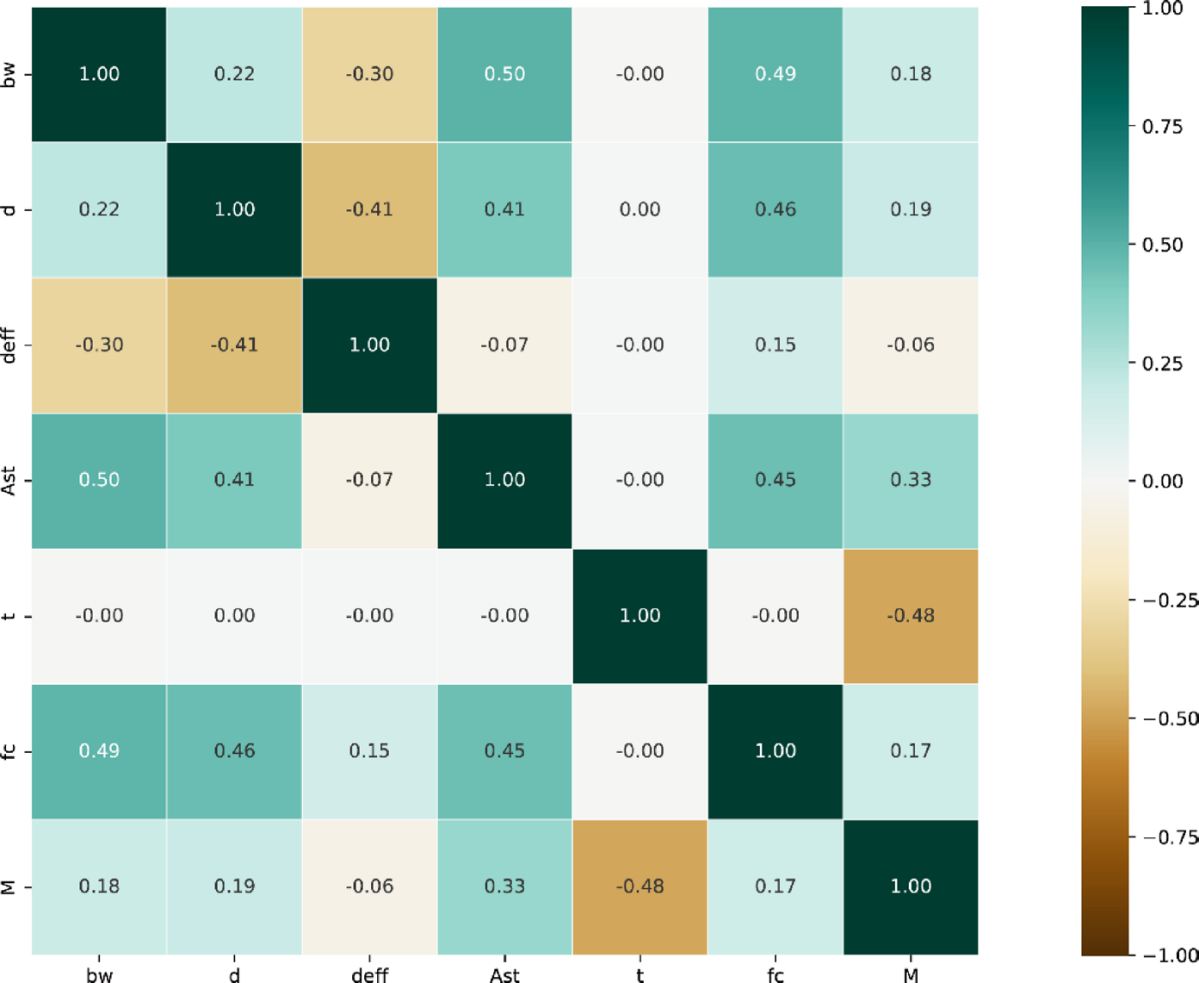
Correlation coefficient plot for input and output parameters.

The time duration of fire loading, $\:t$, is considered over a wide range from zero to 120 min. This range is adequate for evaluating fire effects on RC beams. The area of steel reinforcement $\:{A}_{st}$, also covers a wide range suitable for RC beams. Figure 3 shows the correlation coefficients between the input and output parameters. It is evident that the time duration of fire $\:t$ and the moment capacity$\:\:{M}_{r}$, which has a correlation coefficient of -0.48, exhibit the strongest correlation among the parameters. This indicates a low correlation and an inverse relationship between fire duration and the beam’s moment capacity. Following this, the steel reinforcement parameter $\:{A}_{st}$ has a correlation coefficient of + 0.33, showing a positive but low correlation with the flexural capacity of the RC beam. The correlation coefficients of the remaining input parameters with the moment capacity are all below 0.35. This demonstrates that there is no strong linear relationship between these variables and the RC beam moment capacity, and more complex nonlinear modeling is required. Most nonlinear machine learning methods are capable of establishing suitable nonlinear relationships between the input and output variables.

One of the reasons for using machine learning (ML) methods and the GEP method is to derive a simple nonlinear mathematical equation capable of describing complex physical models, such as RC beams under fire loading. In the following, the GEP model is employed to identify this nonlinear relationship. As a white-box model, it can mathematically and analytically provide an explicit relationship to predict the moment capacity of an RC beam under fire loading. Black-box machine learning models are also applied to accurately capture the nonlinear relationship and to compare their performance with that of the GEP model.

## Methodology

**Fig. 4:**
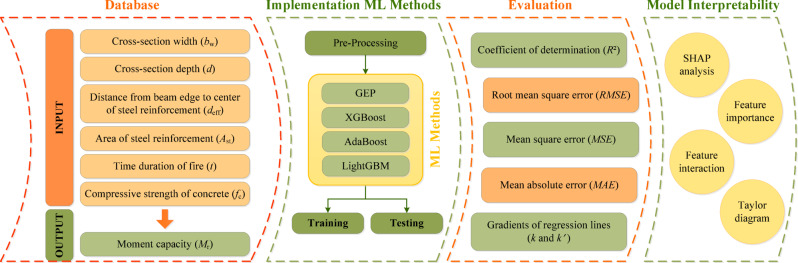
The flowchart of the methodology.

The present study introduces a novel machine learning framework that addresses critical gaps in research on fire-exposed reinforced concrete (RC) structures. This methodology integrates advanced ML techniques within a rigorously defined workflow, as illustrated in Fig. 4. The moment capacity of RC beams under fire conditions is predicted using the GEP, XGBoost, AdaBoost, and LightGBM models. The database for estimating *M*_r_ of RC beams comprises 280 samples. The cross-section width$\:{\:b}_{w}$, cross-section depth $\:d$, distance from the beam edge to the center of steel reinforcement $\:{d}_{eff}$, area of steel reinforcement $\:{A}_{st}$, time duration of fire $\:t$, compressive strength of concrete $\:{f}_{c}$, and moment capacity of the beam under fire $\:{M}_{r}$ are set as the ML method parameters. The statistical characteristics of each parameter, including the mean, standard deviation, kurtosis, skewness, minimum, and maximum values, are calculated. Model performance is evaluated using statistical metrics such as the coefficient of determination (*R*^2^), root mean square error (*RMSE*), mean square error (*MSE*), mean absolute error (*MAE*), and the gradients of regression lines (*k* and *k*′). SHAP analysis is applied to interpret the predictions of the XGBoost model, and the mean |SHAP| values and feature importance for each parameter are also calculated. In addition, a Taylor diagram is employed to assess the accuracy and error of the ML models.

### GEP model development

**Fig. 5:**
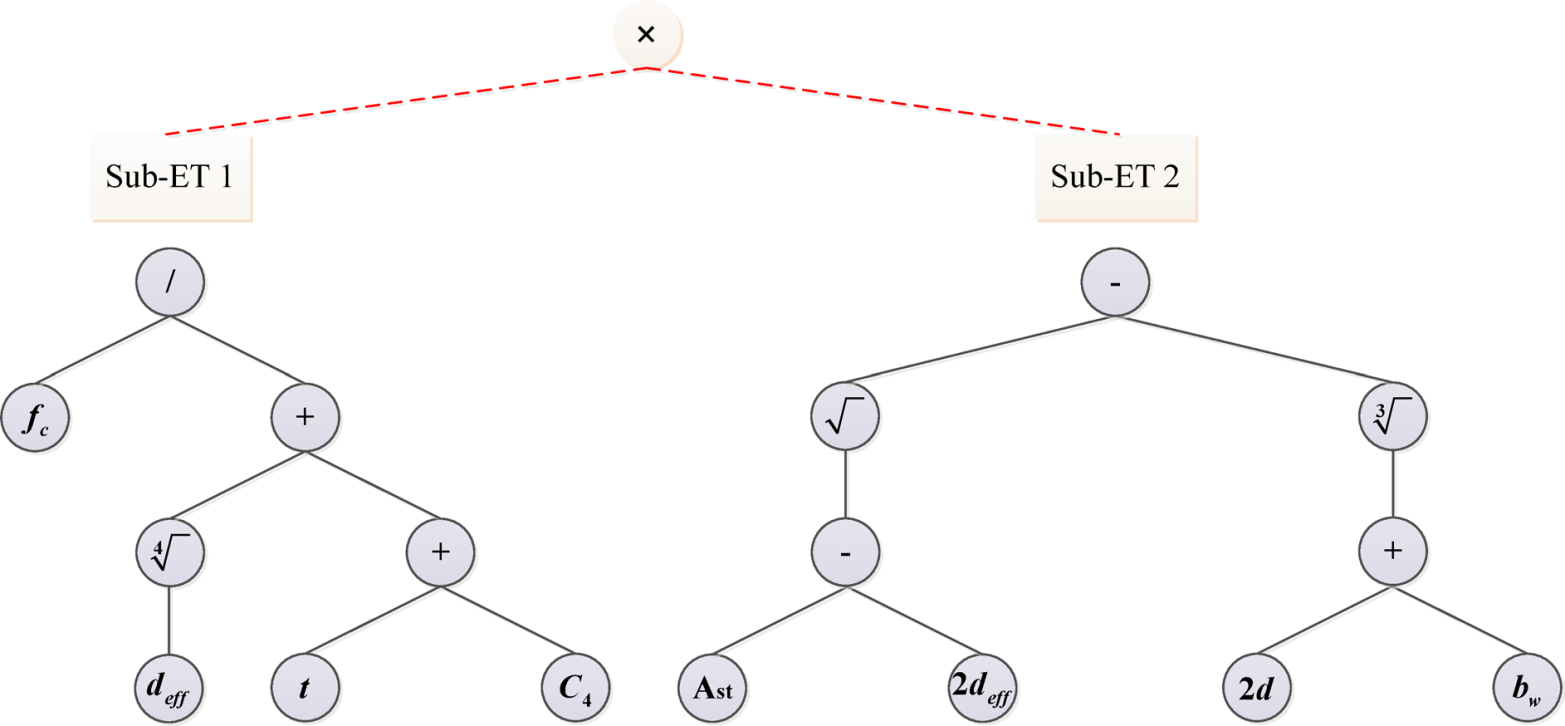
Expression tree of a new formulation for RC beam moment capacity.

The Gene Expression Programming (GEP) approach was originally proposed by Ferreira et al.^44^ as an enhancement of genetic algorithms (GA). This method demonstrates superior efficiency compared to both standard genetic algorithms and genetic programming (GP), a machine learning approach initially developed by Cramer and later enhanced by Koza. GEP employs linear chromosome structures that serve as genotypes, which are then expressed as parse tree phenotypes through complex expression trees (ETs). In the GEP model, chromosomes are organized as expression trees, requiring specialized interpretation. To decode these tree-structured genetic codes, researchers developed Karva, a specialized translation language. A distinctive feature of this methodology is its use of the Karva language, specifically designed to interpret the encoded chromosomal information. In this study, the advanced GEP technique is used to estimate the moment capacity of RC beams under fire conditions. A new formulation for calculating the moment capacity of RC beams is developed using the GEP method. The parameter settings for the GEP approach are summarized in Table 2. Figure 5 presents the expression tree decomposition of the proposed moment capacity formula for RC beams.

**Table 2:** Optimal values of parameters for GEP.

Category	Parameter	Value
Genetic operators	Inversion	0.00
	Permutation	0.00
	Best Cloning	0.0716
	RNC Mutation	0.0328
	Constant Fine-Tuning	0.0728
	Constant Range Finding	0.00011
	Gene Recombination	0.00
	Dc Inversion	0.014
	Dc Permutation	0.014
	Function Insertion	0.00
	Random Cloning	0.0132
General	Chromosomes	1000
	Genes	2
	Head Size	5
	Tail Size	16
	Dc Size	16
	Gene Size	37
Numerical Constants	Constants per Gene	10
	Data Type	Integer
	Lower Bound	− 10
	Upper Bound	10
Data	Independent Variables	6
	Training Records	220
	Validation Records	60
Program Structure	Program Size	19
	Literals	9

The new independent formulation for the moment capacity of RC beams is presented in Eq. (9). Unlike the complexity of many other equations, this formulation can be easily applied in manual calculations, engineering software, or programming applications. Therefore, it is practical for real-world use. In addition, this relationship effectively captures the interactions between variables, including the inverse relationship with fire duration and the direct relationship with compressive strength.

9 $${M_r}=\frac{{{f_c}\left( {\sqrt {{A_{st}} - 2{d_{eff}}} - \sqrt[3]{{2d+{b_w}}}} \right)}}{{\sqrt[4]{{d^{\prime}}}+t+0.864}}$$

**Fig. 6:**
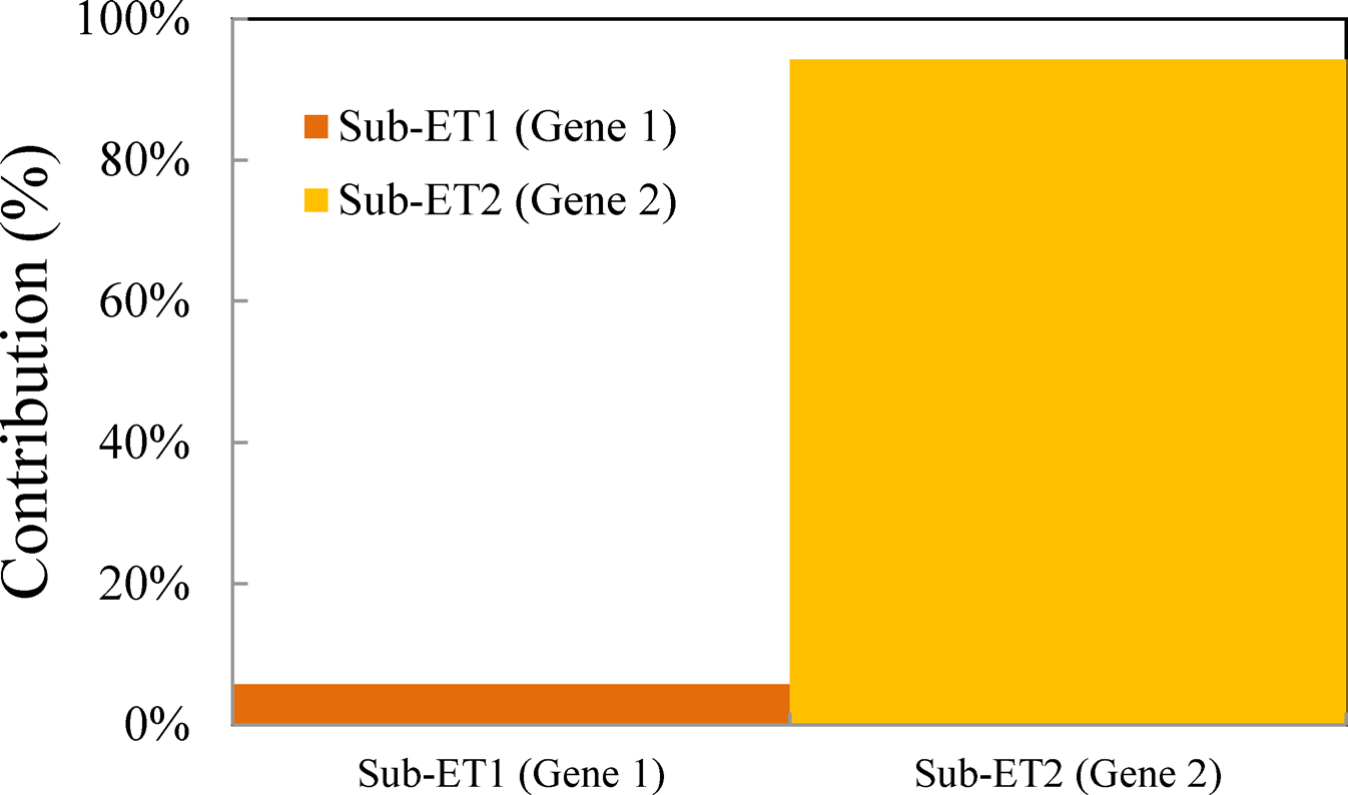
Contribution of individual genes.

The contribution of each gene was calculated as the mean absolute value of its output across the entire test dataset^42^. The percentage contribution was then determined relative to the total values of all terms. The results, shown in Fig. 6, clearly illustrate the percentage contribution of each gene. Gene 2 is the dominant term, contributing approximately 94.2% to the model’s prediction, while Gene 1 has a smaller influence, accounting for 5.8%.

### Machine learning methods

#### Hyperparameter tuning

Hyperparameter optimization is a critical step in machine learning for fine-tuning model parameters to achieve optimal performance. Unlike traditional methods, the Bayesian optimization approach intelligently explores the hyperparameter space, reducing computational cost while improving accuracy^43^. In this study, the ML framework is implemented using a Bayesian optimization approach.

Bayesian Optimization (BO)^44^ offers a more efficient alternative to conventional methods and is employed in this study to optimize hyperparameter settings^45^. BO enhances the search for optimal configurations by modeling the prior distribution of the objective function. The method begins by defining the hyperparameter ranges and initial distributions for each model, with the ultimate goal of maximizing performance within these ranges. By eliminating unproductive experiments, BO facilitates the identification of the most efficient hyperparameter combinations and significantly improves the efficiency of the tuning process^46^. The hyperparameters of the XGBoost, AdaBoost, and LightGBM models were optimized using BO with a Gaussian Process as a surrogate model. The procedure was implemented using the scikit-learn library in Python. The objective of the optimization was to minimize the mean squared error (MSE) from a 5-fold cross-validation on the training dataset, with this cross-validated score serving as the objective function for the BO algorithm. For each model, 35 BO iterations were performed, a number chosen to provide a robust search of the hyperparameter space while remaining computationally feasible. The hyperparameter prior distributions are provided in Tables 3 and 4, and 5.

Parameters optimized during this process included the number of columns sampled by each tree, learning rate, maximum depth, number of estimators, subsample ratio of training instances, and random state.

#### XGBoost

eXtreme Gradient Boosting (XGBoost) was developed to enhance speed and efficiency. It is a boosting method for machine learning, initially created by Tianqi Chen^47^ and subsequently expanded by a diverse group of developers. XGBoost is an improved version of Gradient Boosting and belongs to ensemble learning methods, which combine multiple weak models to create a stronger predictive model. XGBoost uses decision trees as base learners, combining them sequentially to improve model performance. The boosting process is progressive, minimizing errors generated by preceding trees. In addition, XGBoost supports parallel processing, handles missing data, and provides multiple parameters for customization, making it suitable for complex and large datasets. In this study, the optimal parameter values for the XGBoost model are listed in Table 3.

**Table 3:** Optimal values of parameters for XGBoost.

Parameter	Prior distribution	Value
Colsample bytree	Uniform	0.67336
Learning rate	Log-Uniform	0.09823
Max depth	Integer Uniform	6
N estimators	Integer Uniform	146
Subsample	Uniform	0.71649
Random state	–	42

#### AdaBoost

Adaptive Boosting (AdaBoost) is a reinforcement learning algorithm used to enhance the performance of weak models^48^. AdaBoost works by combining multiple weak models to create a stronger predictive model. In this algorithm, a weak model is first trained on the data. Examples that are incorrectly predicted at this stage are assigned higher weights. In the next stage, a new weak model is trained with greater focus on these high-weight examples. This process is repeated over several iterations. Finally, the outputs of all weak models are combined, with appropriate weighting, to produce the final prediction.

**Table 4:** Optimal values of parameters for AdaBoost.

Parameter	Prior distribution	Value
N estimators	Integer Uniform	200
Learning rate	Log-Uniform	0.067502
Max depth	Integer Uniform	9

#### LightGBM

Light Gradient Boosting Machine (LightGBM)^49^ is an advanced algorithm developed by Microsoft, designed to train reinforcement models faster and more efficiently. Like other boosting methods, it sequentially trains weak models, usually decision trees, and combines them to produce a final model with higher accuracy. However, LightGBM offers significant improvements in speed and memory efficiency. One of its major advantages is very high training speed, achieved through techniques such as histogram-based decision trees and gradient-based one-side sampling (GOSS), which reduce the volume of processed data without compromising accuracy. LightGBM employs a histogram-based algorithm combined with a leaf-wise tree growth strategy with a specified maximum depth. It divides the continuous values of each feature into discrete bins and creates histograms by counting the samples in these bins, which greatly accelerates the search for the optimal split points^50^. The optimal parameter values for LightGBM are summarized in Table 5.

**Table 5:** Optimal values of parameters for LightGBM.

Parameter	Prior distribution	Value
Colsample bytree	Uniform	0.76660
Learning rate	Log-Uniform	0.24843
Max depth	Integer Uniform	8
N estimators	Integer Uniform	102
Subsample	Uniform	0.86294
Random state	–	42

## Results and discussions

To evaluate the predictive performance of the beam moment capacity models more rigorously, several statistical measures were employed: the coefficient of determination (*R*^2^), which indicates the proportion of variance in the experimental data explained by each model; the root mean square error (*RMSE*)^51^, which measures the average magnitude of prediction errors; the mean square error (*MSE*); the mean absolute error (*MAE*); and the gradients of regression lines (*k* and *k*′)^52–54^. The formulations of these quantitative statistical indices are provided in Table 6.

**Fig. 7:**
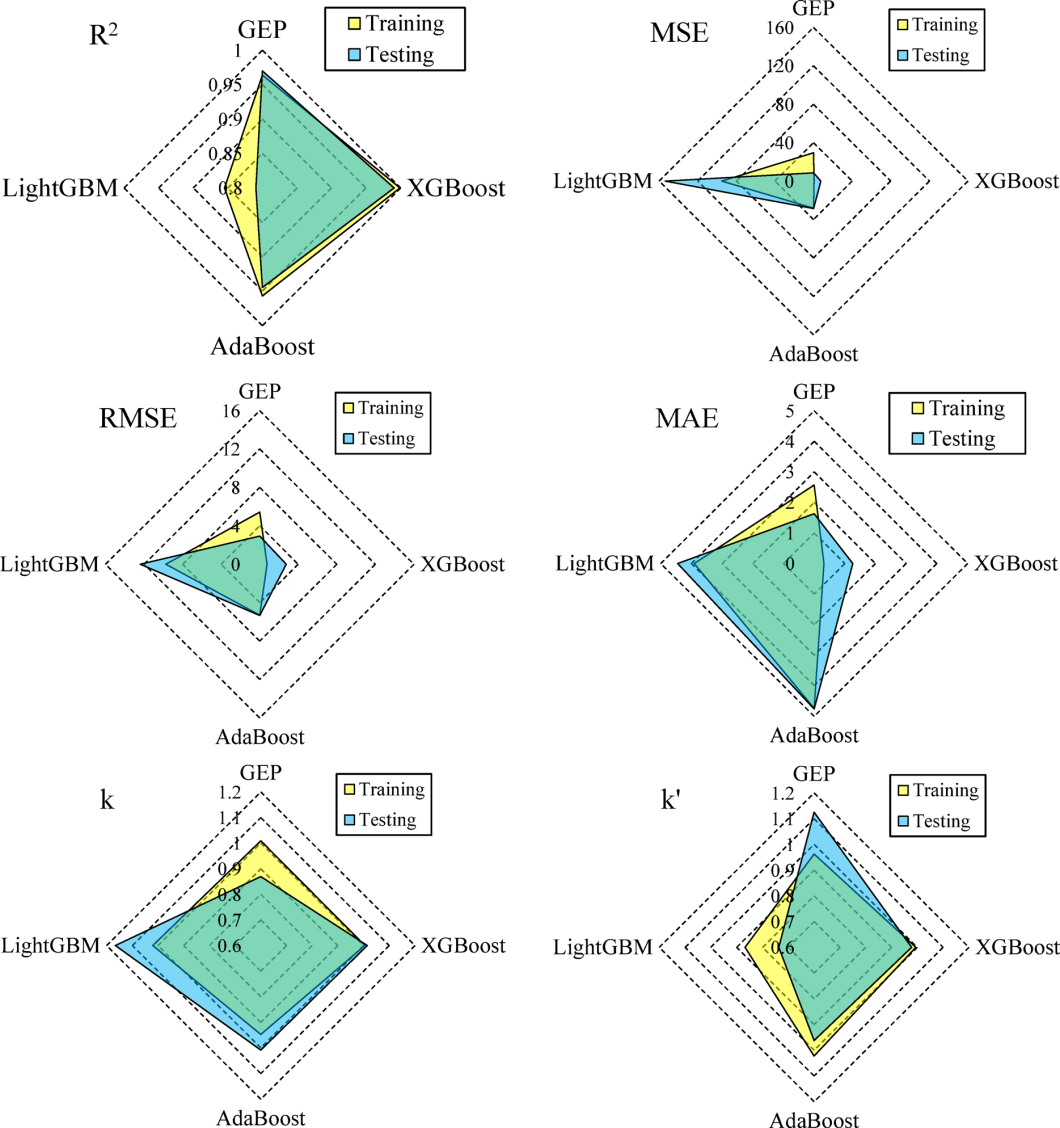
Radar chart comparing the performance metrics of GEP, XGBoost, AdaBoost, and LightGBM models.

The comparative performance analysis of GEP, XGBoost, AdaBoost, LightGBM, and a reference ANN model^30^ provides critical insights into their predictive capabilities for moment capacity, as summarized in Table 7. Figure 7 presents a radar chart comparing the performance metrics (*R*^2^, *RMSE*, *MAE*, *MSE*, *k* and *k*′) of the GEP, XGBoost, AdaBoost, and LightGBM models in predicting the moment capacity of RC beams under fire conditions.

**Table 6:** Formulation of equations for statistical indices.

Statistical index	Formulation of parameter	Number of equation
*R* ^*2*^	$\frac{{{{\left( {\sum\limits_{{i=1}}^{n} {({M_{r,i,real}} - {{\bar {M}}_{r,real}})({M_{r,i,\boldsymbol{mode}l}} - {{\bar {M}}_{r,\boldsymbol{mode}l}})} } \right)}^2}}}{{\sum\limits_{{i=1}}^{n} {{{({M_{r,i,real}} - {{\bar {M}}_{r,real}})}^2}\,\sum\limits_{{i=1}}^{n} {{{({M_{r,i,\boldsymbol{mode}l}} - {{\bar {M}}_{r,\boldsymbol{mode}l}})}^2}} } }}$	(10)
*MSE*	$\frac{{\sum\limits_{{i=1}}^{n} {{{({M_{r,i,real}} - {M_{r,i,\boldsymbol{mode}l}})}^2}} }}{n}$	(11)
*RMSE*	$\sqrt {\frac{{\sum\limits_{{i=1}}^{n} {{{({M_{r,i,real}} - {M_{r,i,\boldsymbol{mode}l}})}^2}} }}{n}}$	(12)
*MAE*	$\frac{{\sum\limits_{{i=1}}^{n} {\left| {{M_r}_{{,i,real}} - {M_{r,i,\boldsymbol{mode}l}}} \right|} }}{n}$	(13)
*k*	$\frac{{\sum\limits_{{i=1}}^{n} {{M_r}_{{,i,real}}{M_{r,i,\boldsymbol{mode}l}}} }}{{\sum\limits_{{i=1}}^{n} {M_{{r,i,\boldsymbol{mode}l}}^{2}} }}$	(14)
*k*′	$\frac{{\sum\limits_{{i=1}}^{n} {{M_r}_{{,i,real}}{M_{r,i,\boldsymbol{mode}l}}} }}{{\sum\limits_{{i=1}}^{n} {M_{{r,i,real}}^{2}} }}$	(15)

### Model performance

The GEP model demonstrates a well-balanced approach, combining mathematically transparent expression trees with robust predictive performance, as reflected in its testing metrics (R^2^ = 0.964, MAE = 2.378). In contrast, XGBoost emerges as the most accurate model, achieving superior testing performance (R^2^ = 0.9906, RMSE = 2.75) and exhibiting minimal overfitting, as indicated by the small difference between training and testing results (ΔR^2^ = 0.0085). AdaBoost shows strong consistency across datasets, with a testing R^2^ of 0.9646 compared to a training R^2^ of 0.9570 and moderate error rates (MAE = 4.76), highlighting its suitability for applications with limited training data.

**Table 7:** Statistical index values for each model.

Parameter	GEP		XGBoost		AdaBoost		LightGBM		ANN^30^
	Training	Testing	Training	Testing	Training	Testing	Training	Testing	
*R* ^2^	0.964	0.97	0.9991	0.9906	0.957	0.9646	0.8548	0.8094	0.998
*MSE*	29.49	8.60	0.57	7.56	28.15	28.52	95.12	153.67	1.64
*RMSE*	5.43	2.93	0.76	2.75	5.31	5.34	9.75	12.40	1.28
*MAE*	2.57	1.63	0.32	1.26	4.74	4.76	3.91	4.45	0.839
*k*	1.0092	0.8693	1.0052	1.0146	0.9474	1.0086	1.0206	1.1662	0.1870
*k*′	0.9628	1.1254	0.9942	0.9781	1.0227	0.9624	0.8676	0.7368	0.1871

In contrast, LightGBM demonstrates a relatively lower performance with a testing R^2^ of 0.8094 and an RMSE of 12.40. These findings indicate that XGBoost remains the optimal choice for applications where precision is critical, whereas GEP is preferable when analytical interpretability is essential^55^.

**Fig. 8:**
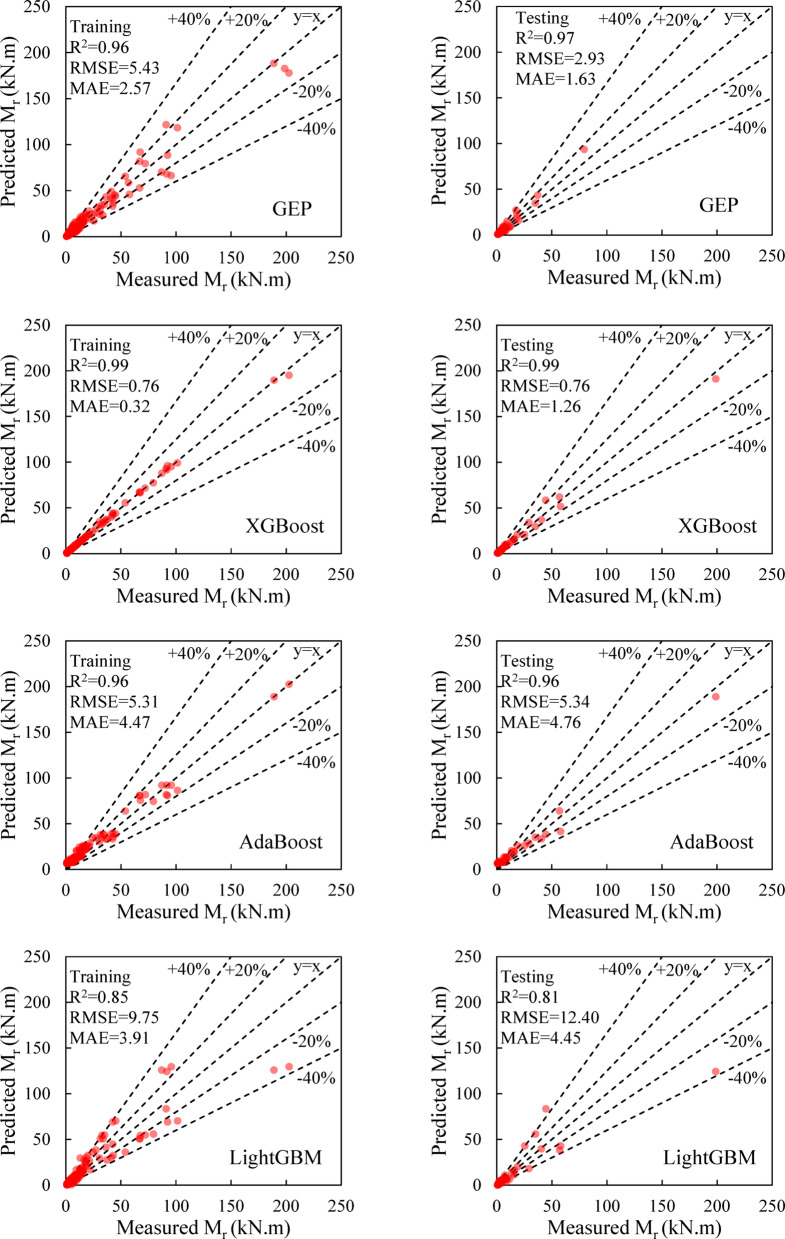
Predicted and measured moment capacitis for training and test phase of the models.

Figure 8 presents 45-degree plots of the predicted moment capacity by GEP and other ML models versus the measured values for both training and testing datasets. The training data contain more points within the ± 20% error range compared to the testing data. However, the coefficient of determination is R^2^ = 0.96 for the training data and R^2^ = 0.97 for the test data, indicating that the majority of points lie close to the y = x line. Considering that the training dataset consists of 222 samples and the test dataset of 58 samples, the number of points outside the ± 20% range is relatively small. Furthermore, none of the predicted points fall outside the ± 40% range, demonstrating that the derived mathematical relationship is reasonably accurate.

**Fig. 9:**
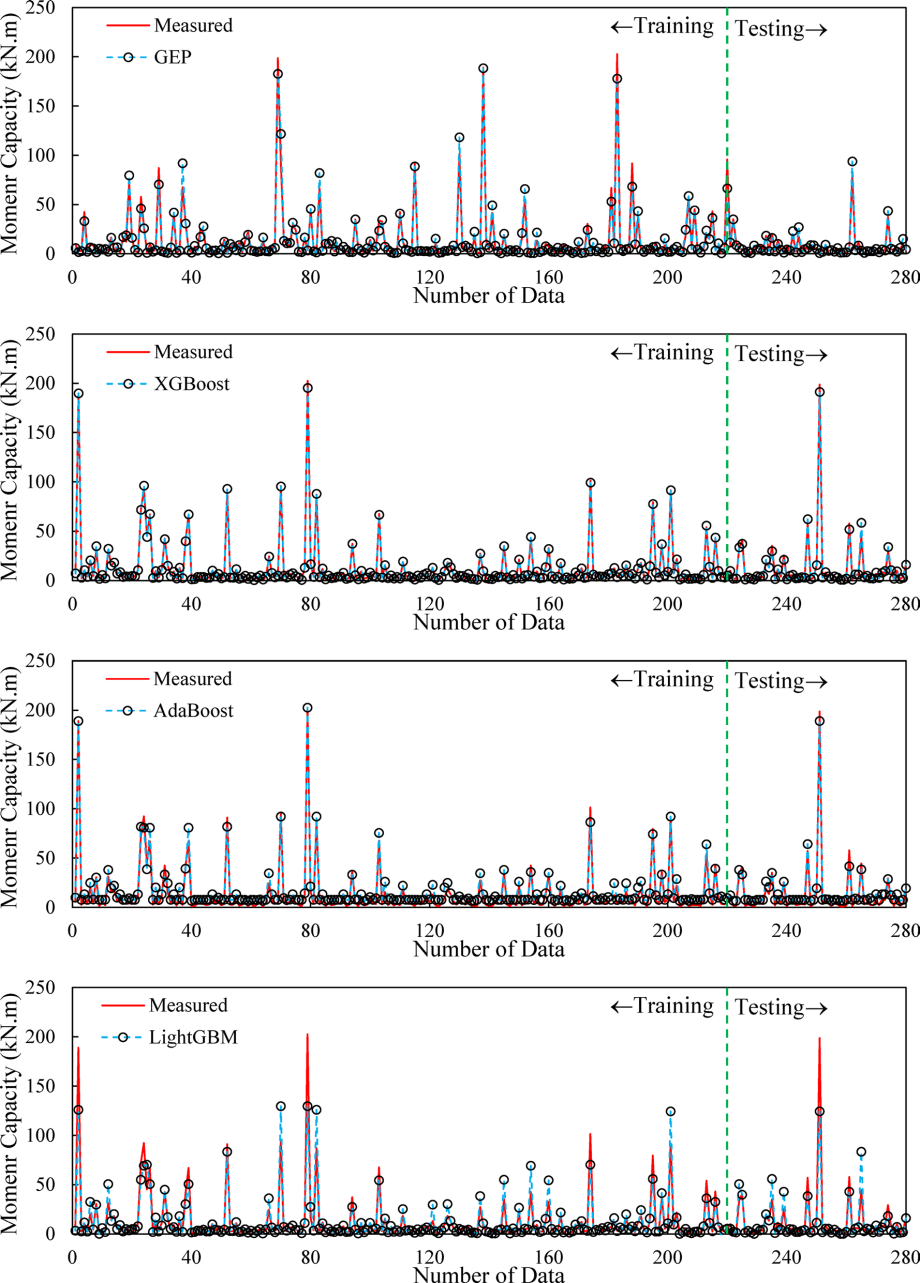
Comparison between measured and predicted history records of the samples.

It should be pointed out that deriving a mathematical relationship with appropriate accuracy for this range of input parameters is a challenging task, and the GEP model has been able to achieve this low error with satisfactory accuracy. However, the XGBoost model exhibits a significantly lower error rate than the other models. Although this model demonstrates high accuracy, it lacks a practical relationship for predicting the moment capacity of the RC beam and is more difficult to interpret compared to mathematical models. The data history for the measured and predicted values by different models is shown in Fig. 9.

According to Fig. 9, despite considerable fluctuations in the data, the model still manages to predict the values accurately. This is because if the model were unable to match points correctly, it would not adequately capture the peaks in this figure. The trend observed in the training data is also present in the test data. To demonstrate that the trend produced by this model is not merely due to complex fluctuations, its behavior should also be evaluated using parametric analysis. The differences between the measured data and the estimated values from the GEP and XGBoost models are well captured. To ensure that the model values are well fitted, the model error is also assessed.

### Error evaluation

**Fig. 10:**
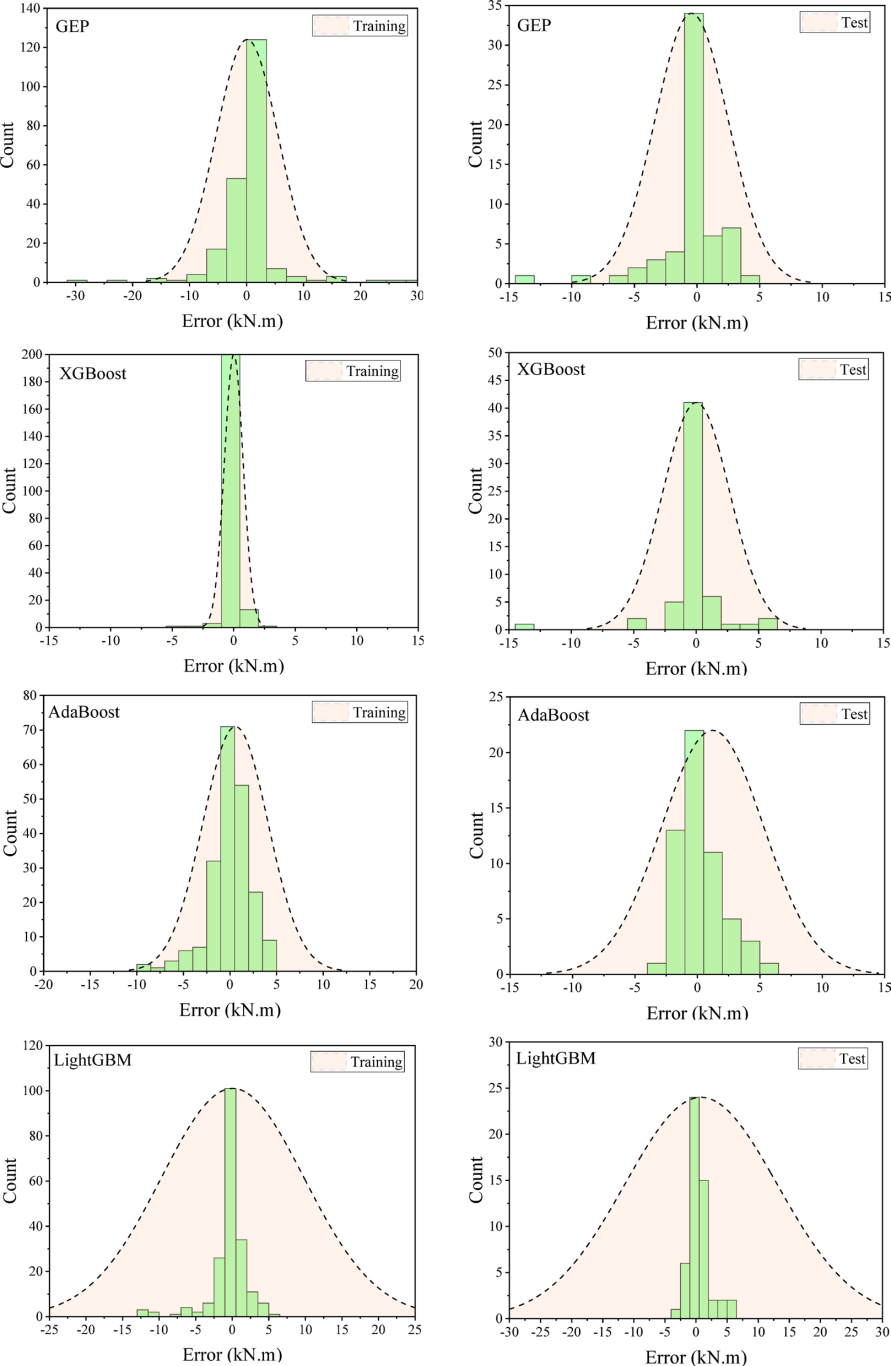
Error histogram for training and test moment capacities of GEP model.

The difference between the predicted and measured data is calculated, and the histogram of the error for the training and test data for different models is shown in Fig. 10. The normal distribution of the error is also overlaid on the bar data. As can be seen, the error distribution for most of the data is concentrated around zero, with the zero-error point located at the center of the curve.

To ensure the normality of the error data distribution in both the training and testing stages, the Kolmogorov-Smirnov and Anderson-Darling statistical tests were applied^56,57^. Both tests are suitable for small statistical populations and are similar, although the Anderson-Darling test places more weight on the data in the tails of the distribution. A P-value of 0.05 was considered as the significance level for these tests^58,59^. For the GEP model, the P-values for the Kolmogorov-Smirnov and Anderson-Darling tests were 0.089 and 0.066, respectively, which are greater than 0.05, confirming the normality of the error data. For the XGBoost model, the P-values were 0.34 and 0.32, respectively. For the AdaBoost model, the P-values were 0.075 and 0.069, and for the LightGBM model, they were 0.111 and 0.120. As shown by these results, all residuals follow a normal distribution. Since the residuals, or errors, are normally distributed, the selected models are well fitted.

### Model interpretability

#### Global interpretation and feature importance

**Fig. 11:**
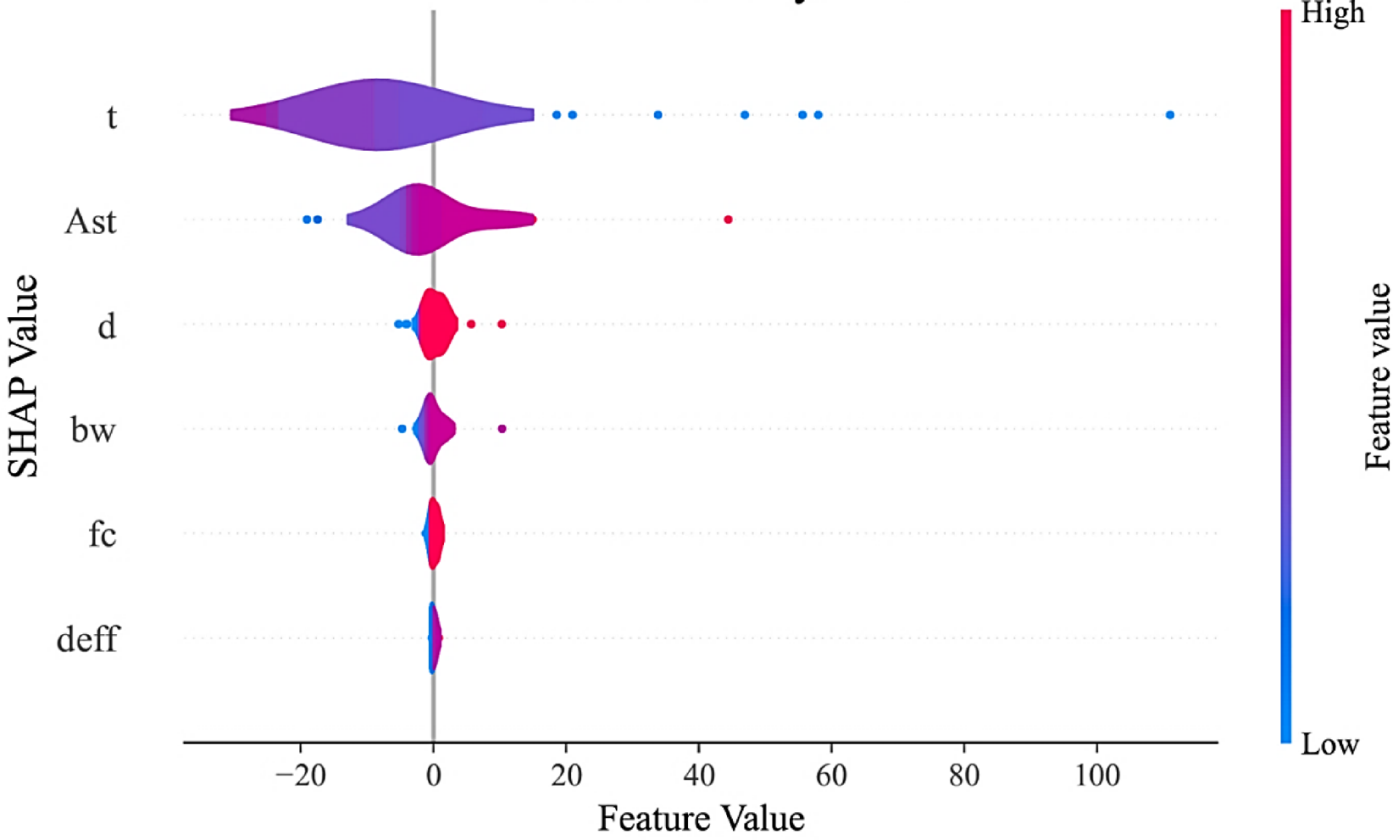
Summary plot of SHAP for XGBoost model.

In ML models, which are black-box in nature, it is not straightforward to study the effect of each parameter and the interactions between variables. For this purpose, SHAP analysis is employed in this study to examine the interpretability of ML models^60,61^. Figure 11 shows a summary plot for SHAP. Because the XGBoost model exhibited high accuracy and low error in predicting the data, SHAP analysis was conducted for this model. In this violin-type figure, the X-axis represents the SHAP value, and the Y-axis represents the problem variables ranked by importance. The time variable $\:t$ and the area of steel reinforcement $\:{A}_{st}$ have the greatest impact on the problem. The $\:{d}_{eff}$ and $\:{f}_{c}$ values have the least impact, while $\:d$ and $\:{b}_{w}$ fall in the intermediate range. It is evident that the duration of the beam’s exposure to fire has a negative impact on the response. The elongation of the violin plot for the area of steel reinforcement, $\:{A}_{st}$, indicates the positive impact of this parameter.

**Fig. 12:**
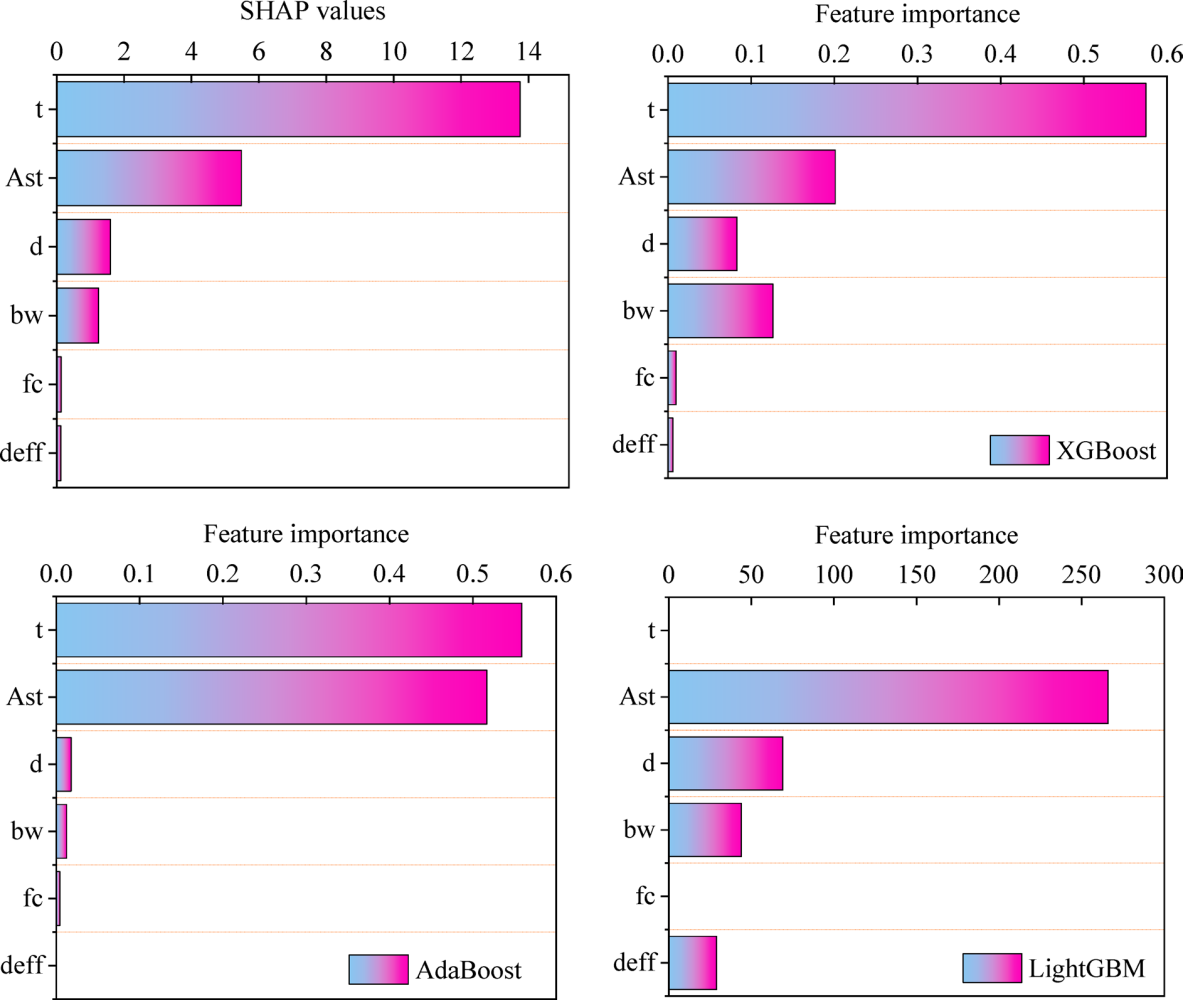
Feature importance for SHAP, XGBoost, AdaBoost and LightGBM models.

Figure 12 shows the $\:mean\left|SHAP\right|$ value for each parameter in the XGBoost model, along with the feature importance graphs calculated internally by each ML model. The $\:mean\left|SHAP\right|$ values obtained from SHAP analysis and the feature importance values from XGBoost are similar for all variables, with the exception of the $\:{b}_{w}$ parameter, which has a slightly higher value in the internal XGBoost calculation. The difference is not significant, suggesting that both methods accurately reflect the effect of each parameter on the response. The AdaBoost model exhibits a similar trend to the SHAP analysis, except that the effect of $\:{A}_{st}$ is greater in AdaBoost than indicated by SHAP. In the LightGBM model, the time variable $\:t$ is not reported as one of the influential variables, which indicates the inability to correctly predict the effect of the variable in the internal calculations of LightGBM. For this reason, SHAP analysis can be considered a reliable analysis for predicting the effect of variables on the problem.

The observed variations in feature importance rankings across different models, and between SHAP and built-in methods, arise from their fundamentally different calculation mechanisms. Built-in importance measures, such as Gini impurity reduction in tree-based models, assess a feature’s contribution based on its ability to homogenize nodes during model training. In contrast, SHAP values use game-theoretic principles to quantify a feature’s average marginal contribution across all possible feature combinations, making them more sensitive to interaction effects. Moreover, model-specific architectures, such as the heightened randomness in Extremely Randomized Trees or the ordered boosting in LightGBM, introduce unique biases in how each algorithm perceives and prioritizes features. These methodological and structural differences naturally lead to divergent importance rankings, even when applied to the same dataset^62^.

**Fig. 13:**
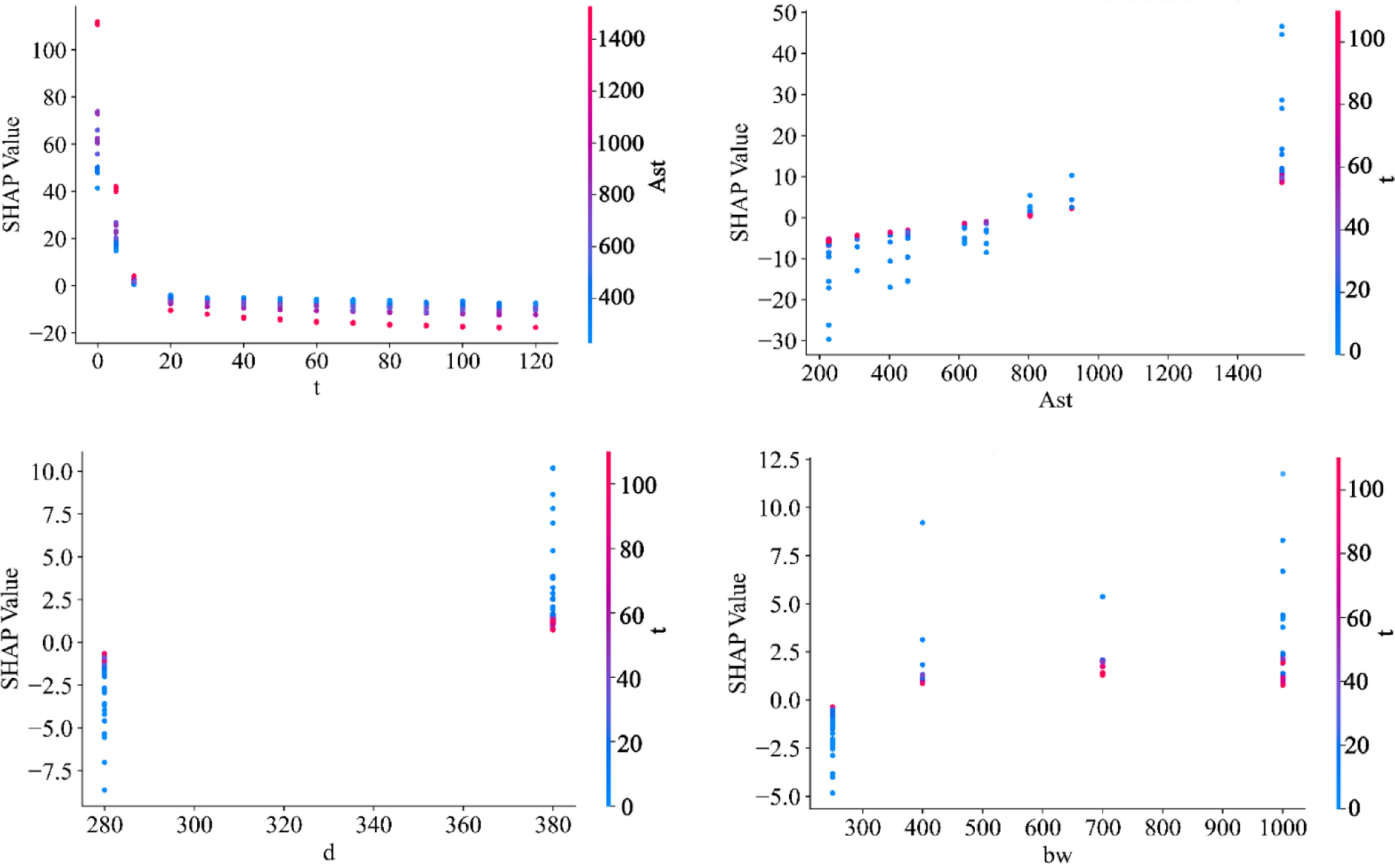
SHAP dependence plot for important variables.

Figure 13 shows the interaction between the variables of fire loading duration t, steel reinforcement area $\:{A}_{st}$, depth $\:d$, and width $\:{b}_{w}$ for the XGBoost model using SHAP analysis. Considering the changes in $\:{A}_{st}$ over time, it is clear that the largest SHAP value was estimated at time $\:t=0$, which decreases after the fire loading period, owing to the reduction in material strength caused by temperature. A similar trend was observed in the study^30^, where the section’s moment capacity decreased significantly after 20 min as the fire loading period increased. It can also be seen that, with increasing steel reinforcement area, the SHAP value increases, which is attributed to the rise in tensile stress in the RC beam section. At time zero, increasing the $\:{A}_{st}$ area has a relatively high influence on raising SHAP values, but as the fire loading duration increases, the effect of $\:{A}_{st}$ on the SHAP value diminishes. This finding is also consistent with the results of Erdem^30^.

In evaluating the effect of depth $\:d$, it is observed that the longer the fire exposure, the smaller the influence of depth $\:d$ on the SHAP value. Nevertheless, its role in mitigating the effects of fire on the RC beam is beneficial. The effect of $\:{b}_{w}$ on the SHAP value is somewhat more complex than that of the other parameters. At time $\:t=0$, when the fire has not yet affected the section, a larger $\:{b}_{w}$ results in a greater SHAP value. However, as the fire loading duration increases, its effect diminishes, and its effectiveness is drastically reduced within 30 min. This behavior was previously reported in^30^. Among the geometric factors of the beam section, increasing depth d appears to have a stronger influence in reducing the impact of fire on the RC beam. This may also be attributed to an increase in the cross-sectional area exposed to fire, which rises with $\:{b}_{w}$.

### Taylor diagram

**Fig. 14:**
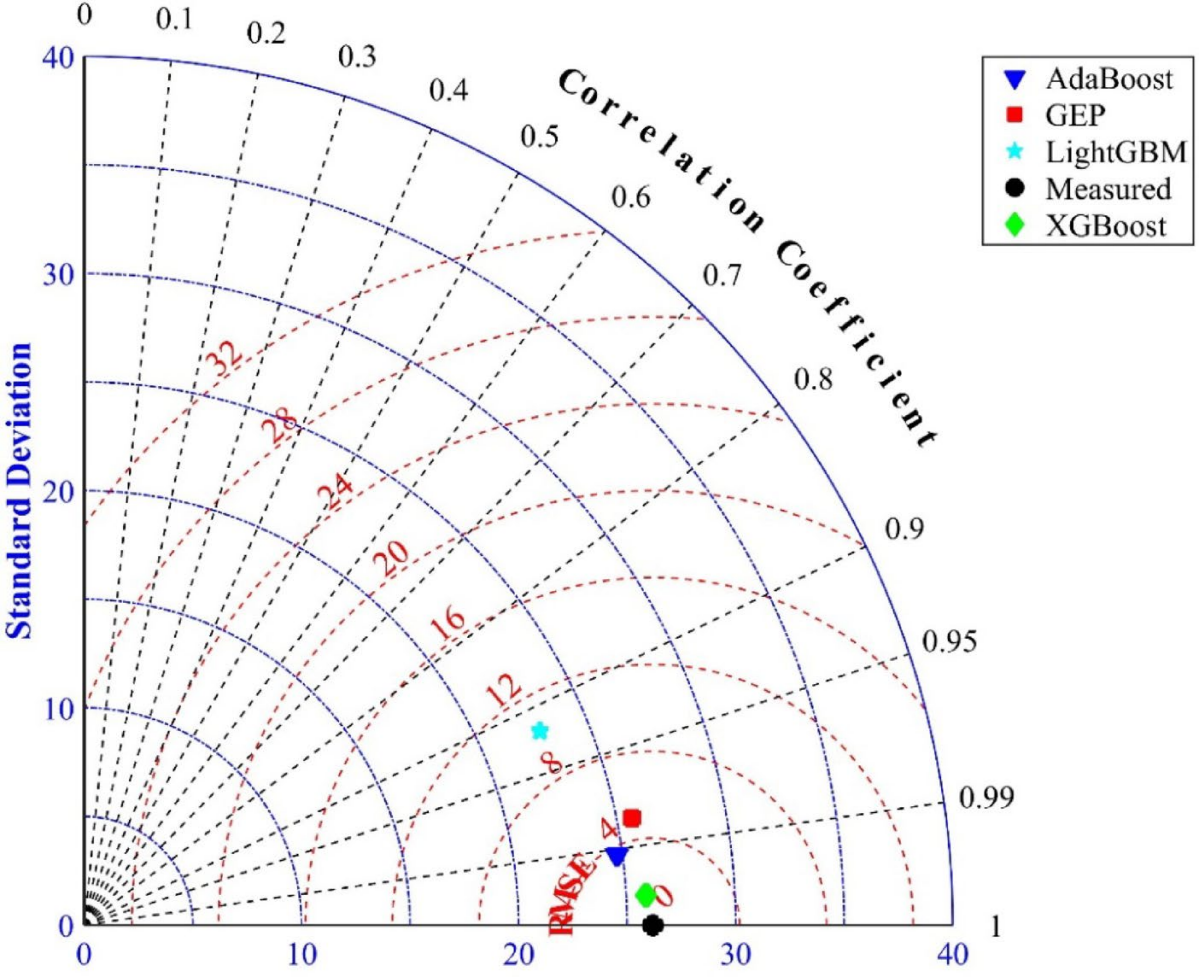
Taylor diagram for different model and measured values.

The Taylor graph is used to evaluate the accuracy and error of ML models. This graph simultaneously displays three parameters: the correlation coefficient, RMSE, and standard deviation. Figure 14 shows the results of the GEP, XGBoost, LightGBM, and AdaBoost models alongside the measured values. The performance of each model in this graph is indicated by how close its points are to the measured data point (black circle). The XGBoost model (green diamond) has the shortest distance to this point, as shown in the figure. The AdaBoost model performs next best, followed by the GEP model. Although the standard deviation values of these two models are nearly identical, the GEP model’s correlation coefficient is lower than that of the XGBoost model. The LightGBM model exhibits the weakest performance. It should be noted that the GEP model remains more practical for applications, even though it is not a black-box model, as it provides a clear and useful mathematical relationship.

## Conclusions

This study investigated the effectiveness of ML methods in estimating the moment capacity of RC beams under fire conditions. Four advanced ML models, GEP, XGBoost, AdaBoost, and LightGBM, were employed. The comprehensive database of 280 samples, with parameters such as cross-section dimensions (*b*_w_, *d*), steel reinforcement details (*d*_eff_, *A*_st_), fire duration (*t*), concrete compressive strength (*f*_c_), and moment capacity (*M*_r_), was considered to evaluate the models. The final results of the study are summarized as follows:

- The main objectives of this study are to implement high-accuracy machine learning models and to provide a very small and practical mathematical equation using the GEP model.
- The models exhibited high predictive accuracy, as validated by statistical metrics (*R*^2^, *RMSE*, *MSE*, *MAE*) and regression gradients (*k*, *k’*).
- The results of SHAP analysis show that increasing the depth *d* has a stronger influence on reducing the effects of fire on the RC beam.
- XGBoost demonstrated the best performance with an R^2^ of 0.9906, outperforming GEP by 2.7% and LightGBM by 18% in prediction accuracy. This model shows a 78% reduction in prediction error (RMSE = 2.75) compared to the RMSE of LightGBM (RMSE = 12.40).
- The results of the Taylor diagram show that the XGBoost model has the best performance, followed by AdaBoost, and then the GEP model. Although the standard deviation values of the two models are nearly identical, the GEP model’s correlation coefficient is lower than that of XGBoost. The LightGBM model shows the weakest performance.

## Data Availability

The datasets generated and/or analyzed during the current study are available from the corresponding author, Younes Nouri (nouri.younes@stu.um.ac.ir), upon reasonable request.

## References

[1] Fire Protection Committee. *Structural Fire Engineering* (American Society of Civil Engineers, 2018). 10.1061/9780784415047.IN

[2] Pachideh, G., Gholhaki, M. & Nouri, Y. An investigation into the impact of fire on lateral stability and strength of thin steel plate shear walls. *Amirkabir J. Civ. Eng.* **52**, 859–872. 10.22060/ceej.2018.15003.5809 (2020).

[3] Buchanan, A. H. & Abu, A. K. Structural design for fire safety: second edition. *Struct. Des. Fire Saf. Second Ed.* 1–415. 10.1002/9781118700402 (2016).

[4] Eftekhar Afzali, S. et al. Compaction and compression behavior of waste materials and Fiber-Reinforced Cement-Treated sand. *J. Struct. Des. Constr. Pract.* **30**, 04025007. 10.1061/JSDCCC.SCENG-1643 (2025).

[5] LaMalva, K. & Hopkin, D. (eds) International Handbook of Structural Fire Engineering (2021). 10.1007/978-3-030-77123-2

[6] Asaad Samani, A. & Hoseini Vaez, S. R. Fire resistance and collapse mechanisms of 2D steel moment frames: A numerical study. *J. Rehabil Civ. Eng.* 0. 10.22075/JRCE.2025.36843.2269 (2025).

[7] Wang, S., Fu, Y., Ban, S., Duan, Z. & Su, J. Genetic evolutionary deep learning for fire resistance analysis in fibre-reinforced polymers strengthened reinforced concrete beams. *Eng. Fail. Anal.* **169**, 109149. 10.1016/J.ENGFAILANAL.2024.109149 (2025).

[8] Liu, C., Liu, X., Yan, L. & Zheng, C. Experimental study on bond behavior of corroded reinforced concrete under coupling effect of fatigue load and elevated temperature. *Eng. Fail. Anal.* **166**, 108862. 10.1016/J.ENGFAILANAL.2024.108862 (2024).

[9] Wang, G., Zheng, Z., Wang, J., Jiang, J. & Lv, Y. Experimental study on fire response of large-scale RC space frame structures and numerical calculation method. *Eng. Fail. Anal.* **171**, 109362. 10.1016/J.ENGFAILANAL.2025.109362 (2025).

[10] Cao, V. & Van, Nguyen, V. N. Flexural performance of postfire reinforced concrete beams: experiments and theoretical analysis. *J. Perform. Constr. Facil*. **36**, 04022029. 10.1061/(ASCE)CF.1943-5509.0001739 (2022).

[11] Yang, J., Yan, K., Doh, J. H. & Zhang, X. Experimental study on shear performance of ultra-high-performance concrete beams at elevated temperatures. *Eng. Struct.* **291**, 116304. 10.1016/J.ENGSTRUCT.2023.116304 (2023).

[12] Noman, M. & Yaqub, M. Restoration of dynamic characteristics of RC T-beams exposed to fire using post fire curing technique. *Eng. Struct.* **249**, 113339. 10.1016/J.ENGSTRUCT.2021.113339 (2021).

[13] Hostetter, H., Naser ·, M. Z., Hawileh, R. A. & Zhou, H. Karaki · Ghada, Enhancing fire resistance of reinforced concrete beams through sacrificial reinforcement. Archit Struct Constr. **2**, 311–322. (2022). 10.1007/S44150-022-00061-W

[14] Banerji, S. & Kodur, V. Numerical model for tracing the response of Ultra-High performance concrete beams exposed to fire. *Fire Mater.* **47**, 322–340. 10.1002/FAM.3099 (2023).

[15] Ren, P., Hou, X., Cui, Z., Xie, H. & Abid, M. Fire resistance evaluation and minimum reinforcement ratio for hybrid fibre-reinforced RPC beams under fire exposure. *J. Build. Eng.* **44**, 103216. 10.1016/J.JOBE.2021.103216 (2021).

[16] Kodur, V. K. R. & Banerji, S. Comparative fire behavior of reinforced concrete beams made of different concrete strengths. *Fire Technol.* **60**, 3117–3146. 10.1007/S10694-023-01375-X/FIGURES/15 (2024).

[17] Qin, H. et al. Experimental research on the spalling behaviour of ultra-high performance concrete under fire conditions. *Constr. Build. Mater.* **303**, 124464. 10.1016/J.CONBUILDMAT.2021.124464 (2021).

[18] Hassan, A., Khairallah, F., Elsayed, H., Salman, A. & Mamdouh, H. Behaviour of concrete beams reinforced using basalt and steel bars under fire exposure. *Eng. Struct.* **238**, 112251. 10.1016/J.ENGSTRUCT.2021.112251 (2021).

[19] Liu, C., Zhou, B., Guo, X., Liu, C. & Wang, L. Analysis and prediction methods for the static properties of reinforced concrete beams under fire. *Structures* **47**, 2319–2330. 10.1016/J.ISTRUC.2022.12.041 (2023).

[20] Zhao, X. Y., Chen, J. X. & Wu, B. An interpretable ensemble-learning-based open source model for evaluating the fire resistance of concrete-filled steel tubular columns. *Eng. Struct.* **270**, 114886. 10.1016/J.ENGSTRUCT.2022.114886 (2022).

[21] Nariman, N. A., Hamdia, K., Ramadan, A. M. & Sadaghian, H. Optimum design of flexural strength and stiffness for reinforced concrete beams using machine learning. *Appl. Sci. 2021*. **11**, 11:8762. 10.3390/APP11188762 8762 (2021).

[22] Khan, M. et al. Intelligent prediction modeling for flexural capacity of FRP-strengthened reinforced concrete beams using machine learning algorithms. *Heliyon* **10**, e23375. 10.1016/J.HELIYON.2023.E23375 (2024). 38169887 10.1016/j.heliyon.2023.e23375 PMC10758834

[23] Solhmirzaei, R., Salehi, H. & Kodur, V. Predicting flexural capacity of Ultrahigh-Performance concrete beams: machine Learning–Based approach. *J. Struct. Eng.* **148**, 04022031. 10.1061/(ASCE)ST.1943-541X.0003320 (2022).

[24] Kumar, R., Rai, B. & Samui, P. Machine learning techniques for prediction of failure loads and fracture characteristics of high and ultra-high strength concrete beams. *Innov. Infrastruct. Solut.* **8**, 1–20. 10.1007/S41062-023-01191-W/FIGURES/10 (2023).

[25] Naser, M. Z. Observational analysis of Fire-Induced spalling of concrete through ensemble machine learning and surrogate modeling. *J. Mater. Civ. Eng.* **33**, 04020428. 10.1061/(ASCE)MT.1943-5533.0003525 (2020).

[26] Naser, M. Z. & Kodur, V. K. Explainable machine learning using real, synthetic and augmented fire tests to predict fire resistance and spalling of RC columns. *Eng. Struct.* **253**, 113824. 10.1016/J.ENGSTRUCT.2021.113824 (2022).

[27] Panev, Y., Kotsovinos, P., Deeny, S. & Flint, G. The use of machine learning for the prediction of fire resistance of composite shallow floor systems. *Fire Technol.* **57**, 3079–3100. 10.1007/S10694-021-01108-Y/FIGURES/19 (2021).

[28] Ye, Z., Hsu, S. C. & Wei, H. H. Real-time prediction of structural fire responses: A finite element-based machine-learning approach. *Autom. Constr.* **136**, 104165. 10.1016/J.AUTCON.2022.104165 (2022).

[29] Environmental, H. E. & TJ of E&. Nominal moment capacity of box reinforced concrete beams exposed to fire. CiteseerH ErdemTurkish J Eng Environ Sci 2009•Citeseer 2009;33:31–44. (2009). undefined 10.3906/muh-0811-4

[30] Erdem, H. Predicting the moment capacity of RC beams exposed to fire using ANNs. *Constr. Build. Mater.* **101**, 30–38. 10.1016/J.CONBUILDMAT.2015.10.049 (2015).

[31] Erdem, H. Prediction of the moment capacity of reinforced concrete slabs in fire using artificial neural networks. *Adv. Eng. Softw.* **41**, 270–276. 10.1016/J.ADVENGSOFT.2009.07.006 (2010).

[32] Ahmadi, M., Kheyroddin, A., Dalvand, A. & Kioumarsi, M. New empirical approach for determining nominal shear capacity of steel fiber reinforced concrete beams. *Constr. Build. Mater.* **234**, 117293. 10.1016/J.CONBUILDMAT.2019.117293 (2020).

[33] Fakharian, P., Rezazadeh Eidgahee, D., Akbari, M., Jahangir, H. & Ali Taeb, A. Compressive strength prediction of Hollow concrete masonry blocks using artificial intelligence algorithms. *Structures* **47**, 1790–1802. 10.1016/j.istruc.2022.12.007 (2023).

[34] Chen, L. et al. Axial compressive strength predictive models for recycled aggregate concrete filled circular steel tube columns using ANN, GEP, and MLR. *J. Build. Eng.* **77**, 107439. 10.1016/j.jobe.2023.107439 (2023).

[35] Kumarawadu, H., Weerasinghe, P. & Perera, J. S. Evaluating the performance of ensemble machine learning algorithms over traditional machine learning algorithms for predicting fire resistance in FRP strengthened concrete beams. *Electron. J. Struct. Eng.* **24**, 47–53. 10.56748/ejse.24661 (2024).

[36] Habib, A., Barakat, S., Al-Toubat, S., Junaid, M. T. & Maalej, M. Developing machine learning models for identifying the failure potential of Fire-Exposed FRP-Strengthened concrete beams. *Arab. J. Sci. Eng.* **2024 5011**, 50:8475–8490. 10.1007/S13369-024-09497-2 (2024).

[37] Ho, T. N. T., Nguyen, T. P. & Truong, G. T. Concrete spalling identification and fire resistance prediction for fired RC columns using machine Learning-Based approaches. *Fire Technol. 2024 603*. **60**, 1823–1866. 10.1007/S10694-024-01550-8 (2024).

[38] Hao, Z. H., Feng, P., Zhang, S. & Zhai, Y. Machine learning for predicting fiber-reinforced polymer durability: A critical review and future directions. *Compos. Part. B Eng.* **303**, 112587. 10.1016/J.COMPOSITESB.2025.112587 (2025).

[39] ISO834. Fire resistance tests elements of building construction Part 1–9: International Standards Organisation, Geneva; (1975).

[40] Eurocode2. *Design of Concrete Structures ENV 1992 Part 1–2: General Rules Structural Fire Design* (European Committee For Standardization, Brussels, 1995).

[41] Incropera, F., DeWitt, D., Bergman, T. & Lavine, A. Fundamentals of heat and mass transfer. (1996).

[42] Kovačević, M., Hadzima-Nyarko, M., Petronijević, P., Vasiljević, T. & Radomirović, M. Comparative Analysis of Machine Learning Models for Predicting Interfacial Bond Strength of Fiber-Reinforced Polymer*-Concrete Comput.* ;**13**:17. 10.3390/COMPUTATION13010017/S1. (2025).

[43] Snoek, J., Larochelle, H. & Adams, R. P. Practical bayesian optimization of machine learning algorithms. *Adv. Neural Inf. Process. Syst.* ;**25**. (2012).

[44] Wu, J. et al. Hyperparameter optimization for machine learning models based on bayesian optimization. *J. Electron. Sci. Technol.* **17**, 26–40. 10.11989/JEST.1674-862X.80904120 (2019).

[45] Isleem, H. F. et al. Analysis of flow dynamics and energy dissipation in piano key and labyrinth weirs using computational fluid dynamics. *Comput. Fluid Dyn. - Anal. Simulations Appl. [Working Title]*. 10.5772/INTECHOPEN.1006332 (2024).

[46] Eltarabily, M. G., Selim, T., Elshaarawy, M. K. & Mourad, M. H. Numerical and experimental modeling of geotextile soil reinforcement for optimizing settlement and stability of loaded slopes of irrigation canals. *Environ. Earth Sci.* **83**, 246. 10.1007/s12665-024-11560-y (2024).

[47] Chen, T. & Guestrin, C. XGBoost: A scalable tree boosting system. *Proc. ACM SIGKDD Int. Conf. Knowl. Discov Data Min.* **13-17-Augu**, 785–794. 10.1145/2939672.2939785/SUPPL_FILE/KDD2016_CHEN_BOOSTING_SYSTEM_01-ACM.MP4 (2016).

[48] CAO, Y. & MIAO Q-G, LIU J-C, G. A. O. L. Advance and prospects of adaboost algorithm. *Acta Autom. Sin*. **39**, 745–758. 10.1016/S1874-1029(13)60052-X (2013).

[49] Ke, G. et al. LightGBM: A highly efficient gradient boosting decision tree. *Adv. Neural Inf. Process. Syst.* ;**30**. (2017).

[50] Liang, W., Luo, S., Zhao, G. & Wu, H. Predicting hard rock pillar stability using GBDT, XGBoost, and LightGBM Algorithms. Math 2020. *Page* **8**, 8:765. 10.3390/MATH8050765 (765 2020).

[51] Nouri, Y., Ghanizadeh, A. R., Safi Jahanshahi, F. & Fakharian, P. Data-driven prediction of axial compression capacity of GFRP-reinforced concrete column using soft computing methods. *J. Build. Eng.* **101**, 111831. 10.1016/j.jobe.2025.111831 (2025).

[52] Fakharian, P., Bazrgary, R., Ghorbani, A., Tavakoli, D. & Nouri, Y. Compressive strength prediction of green concrete with recycled Glass-Fiber-Reinforced polymers using a machine learning approach. *Polym. (Basel).* 17. 10.3390/polym17202731 (2025). 10.3390/polym17202731 PMC12567245 41150272

[53] Nouri, Y., Ghanbari, M. A. & Fakharian, P. Flexural behavior of hybrid GFRP-steel reinforced concrete beam: experimental and explainable artificial intelligence. *Eng. Struct.* **345**, 121565. 10.1016/j.engstruct.2025.121565 (2025).

[54] Ziaie, A., Mehdizadeh, B., Safi Jahanshahi, F., Ahmadi, N. & Ghanizadeh, A. R. Prediction of Liquefaction-Induced lateral displacements using hybrid GBRT and EOA. *J. Soft Comput. Civ. Eng.* 2026;10: (2061). 10.22115/scce.2025.2061

[55] Fakharian, P. et al. Bond strength prediction of externally bonded reinforcement on groove method (EBROG) using MARS-POA. *Compos. Struct.* 349–350. 10.1016/j.compstruct.2024.118532 (2024).

[56] Massey, F. J. Jr The Kolmogorov-Smirnov test for goodness of fit. *J. Am. Stat. Assoc.* **46**, 68–78 (1951).

[57] Anderson, T. W. & Darling, D. A. A test of goodness of fit. *J. Am. Stat. Assoc.* **49**, 765–769 (1954).

[58] Pettitt, A. N. Testing the normality of several independent samples using the Anderson-Darling statistic. *Appl. Stat.* **26**, 156. 10.2307/2347023 (1977).

[59] Steinskog, D. J., Tjøstheim, D. B. & Kvamstø, N. G. A cautionary note on the use of the Kolmogorov–Smirnov test for normality. *Mon Weather Rev.* **135**, 1151–1157. 10.1175/MWR3326.1 (2007).

[60] Lundberg, S. M. & Lee, S. I. A unified approach to interpreting model predictions. Adv. Neural Inf. Process. Syst., vol. - Decem, Neural information processing systems foundation; 2017, 4766–75. (2017).

[61] Shabani Ammari, A. et al. Flexural strengthening of corroded steel beams with CFRP by using the end anchorage: Experimental, numerical, and machine learning methods. *Case Stud. Constr. Mater.* **23**, e04966. 10.1016/j.cscm.2025.e04966 (2025).

[62] Wang, H., Liang, Q., Hancock, J. T. & Khoshgoftaar, T. M. Feature selection strategies: a comparative analysis of SHAP-value and importance-based methods. *J. Big Data*. **11**, 44. 10.1186/s40537-024-00905-w (2024).

